# Structural plasticity enables broad cA_*n*_ binding and dual activation of CRISPR-associated ribonuclease Cdn1

**DOI:** 10.1093/nar/gkaf1524

**Published:** 2026-01-22

**Authors:** Wenxuan Zhang, Jianping Kong, Yuqin Zeng, Yunning Su, Sijun Zhang, Yutao Li, Chunyi Hu, Qihua Chen, Yibei Xiao, Meiling Lu

**Affiliations:** State Key Laboratory of Natural Medicines, School of Pharmacy, China Pharmaceutical University, Nanjing 211198, China; State Key Laboratory of Natural Medicines, School of Pharmacy, China Pharmaceutical University, Nanjing 211198, China; State Key Laboratory of Natural Medicines, School of Pharmacy, China Pharmaceutical University, Nanjing 211198, China; Department of Biochemistry, School of Life Science and Technology, China Pharmaceutical University, Nanjing 211198, China; Department of Biochemistry, School of Life Science and Technology, China Pharmaceutical University, Nanjing 211198, China; Jiangsu Provincial Key Laboratory of Biology and Therapeutics for Immune System-Related Diseases, China Pharmaceutical University, Nanjing 211198,China; State Key Laboratory of Natural Medicines, School of Pharmacy, China Pharmaceutical University, Nanjing 211198, China; Department of Biological Sciences, Faculty of Science, National University of Singapore, Singapore 117543, Singapore; Precision Medicine Translational Research Programme (TRP), Department of Biochemistry, Yong Loo Lin School of Medicine, National University of Singapore, Singapore 117543, Singapore; Department of Neurology, the Second Xiangya Hospital, Central South University, Changsha 410008, China; Clinical Medical Research Center for Stroke Prevention and Treatment of Hunan Province, Department of Neurology, the Second Xiangya Hospital, Central South University, Changsha 410008, China; State Key Laboratory of Natural Medicines, School of Pharmacy, China Pharmaceutical University, Nanjing 211198, China; Chongqing Innovation Institute of China Pharmaceutical University, Chongqing 401135, China; Department of Biochemistry, School of Life Science and Technology, China Pharmaceutical University, Nanjing 211198, China; Jiangsu Provincial Key Laboratory of Biology and Therapeutics for Immune System-Related Diseases, China Pharmaceutical University, Nanjing 211198,China

## Abstract

Prokaryotes have naturally evolved diverse RNA-guided defense systems against viral infections, with the type III CRISPR–Cas systems representing the most intricate. These systems feature accessory proteins activated by cyclic oligoadenylates (cOAs) produced upon target RNA recognition, synergizing with the CRISPR–Cas machinery to defend against exogenous invaders. Typically, each accessory protein is activated by only one specific cOA type. Here, we characterize Cdn1, a type III-B CRISPR accessory protein from *Psychrobacter lutiphocae*, which binds to cA_3_, cA_4_, and cA_6_, but activated by cA_4_ and cA_6_ with different efficacies to catalyze ssRNA cleavage. Combined structural and biochemical analyses reveal that cOA binding triggers dramatic conformational reorganization, including the formation of a dimerization interface of nuclease domains, the emergence of substrate binding cleft, and the reconstruction of a metal-dependent catalytic center essential for RNA cleavage. This dual activation mechanism illustrates evolutionary innovation within CRISPR-associated Rossman-fold nucleases. We propose that such structural plasticity evolved to maximize defensive resilience during microbial competition and horizontal gene transfer, while preserving broad-spectrum antiviral ability. These findings not only elucidate the activation mechanisms of Cdn1 within the type III systems but also underscore the functional complexity and adaptability of CRISPR–Cas ancillary proteins.

## Graphical Abstract

**Graphical Abstract:**
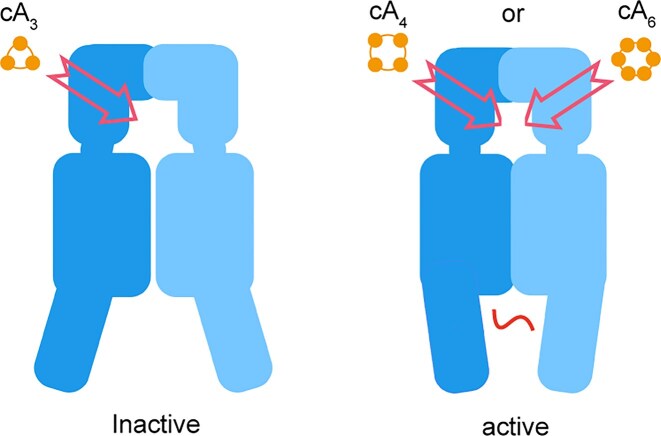

## Introduction

CRISPR–Cas systems are broadly categorized into seven distinct types, each possessing unique mechanisms for targeting foreign genetic elements [1–4]. Among them, type III CRISPR–Cas systems display the most complex interference mechanism. Multiple Cas proteins assemble with CRISPR RNA (crRNA) into interference complexes (Csm complex in type III-A/D systems or Cmr complex in type III-B/C systems) [5–7]. Upon crRNA-guided binding to complementary target nucleic acids, these complexes activate both RNase and DNase activities [8–11]. This activation triggers the Cas10 subunit to synthesize second messengers through polymerizing ATP into cyclic oligoadenylates ranging from 3 to 6 AMPs (cA_3–6_) [5–7, 12–14]. Notably, in the type III system of Bacteroides fragilis, S-adenosyl methionine (SAM)-AMP serves as an exceptional second messenger [15].

A wide array of effector proteins capable of sensing cyclic oligoadenylates (cOAs) have been identified, many of which are genetically linked to CRISPR–Cas loci [16–19]. These effectors typically feature a cOA-binding domain, most commonly the CRISPR-associated Rossmann fold (CARF) or SMODS-associated and fused with various effector domains (SAVED) [18, 19], such as nucleases [20–27], proteases [28], transcription factors [29], deaminases [18, 30, 31], Toll/interleukin-1 receptor (TIR) domains [32], and transmembrane proteins [33]. The induced activation of auxiliary proteins upon cOAs binding is crucial for comprehensive viral clearance, ultimately inducing cellular dormancy or death, resulting in abortive infection that effectively prevents viral propagation [19].

CARF-associated nucleases are among the most extensively characterized CARF-fused effectors [20–27]. Csm6/Csx1-like nucleases possess CARF domains belonging to the CARF1/CARF2 clade [18], and form homo-oligomers with C-terminal higher eukaryotic and prokaryotic nuclease (HEPN) domains. Binding to cOAs activates these HEPN domains to cleave RNAs in a metal-independent manner [20, 21, 27, 34, 35]. The CARF domain in these nucleases not only binds but also hydrolyzes cOAs, thereby mitigating potential self-toxicity [20, 36–39]. The Can2 nuclease family, whose CARF domain belongs to the CARF4 clade and contains PD-(D/E)xK nuclease domains [18, 22, 23], exhibits robust nuclease activity [22–24]. cA_4_ binding activates the dimeric Can2, enabling the cleavage of single-stranded RNA (ssRNA) as well as nicking of double-stranded DNA (dsDNA) or cleavage of single-stranded DNA (ssDNA), although its CARF domain lacks ring nuclease activity [22–24]. Additionally, the EcNucC effector protein from the cyclic oligonucleotide-based antiphage signaling system (CBASS), which includes both SAVED and nuclease domains, is strongly activated by cA_3_ produced by type III CRISPR–Cas systems [40–42]. This detection-activation capability leads to the destruction of both bacterial and phage genome, effectively preventing phage replication [42].

While the activated type III system effector complex produces multiple cOAs, all previously identified effector proteins are activated by only one specific cyclic oligoadenylate. This remained the case until the recent discovery of CARF-fused deaminase effectors belonging to the CARF5 clade, which can be activated by either cA_4_ or cA_6_ with similar efficiency [18, 30, 31]. Whether this sensing plasticity extends to other CARF-fused effectors remains unknown. Here, we present a Can2 family effector protein associated with the type III-B CRISPR–Cas locus of *Psychrobacter lutiphocae*. This endoribonuclease binds to cA_3_, cA_4_, and cA_6_, but is activated by both cA_4_ and cA_6_ with distinct efficacies to catalyze ssRNA cleavage proximal to uridine or adenosine. Furthermore, we determined the cA_3_-bound inactive and cA_4_-bound active structures, revealing that cOA-induced conformational reorganization drives catalytic activation through three coordinated mechanisms: (i) formation of a stable nuclease domain dimer interface; (ii) assembly of a substrate-binding cleft; and (iii) reconstruction of a metal-dependent active site essential for phosphodiester bond hydrolysis.

## Materials and methods

### DNA and RNA oligonucleotides

All the sequences (HPLC purified) used in this study are shown in Supplementary Table S2. The primers were purchased from Sangon Biotech (Shanghai) Co., Ltd (Shanghai, China). The fluoresce labeled substrates and the gene sequence of Cdn1 were synthesized in GenScript. Cyclic oligonucleotides were purchased from BioLog Life Science.

### Plasmids construction

The codon-optimized gene of Cdn1 was inserted into pET-28a (+) vector. The variants were constructed through site-directed mutagenesis. All the constructions were verified by Sanger sequencing (Sangon Biotech).

### Protein expression and purification

*Escherichia coli* BL21(DE3) harboring WT and mutants of Cdn1 was grown in LB broth supplemented with 50 µg/ml kanamycin at 37°C till the OD_600_ reached 0.6. Expression of Cdn1 was induced with the adding of 0.5 mM of isopropylthio-β-d-galactoside (IPTG) and the cells were grown for 12 h at 25°C. Cells were harvested and re-suspended in the lysis buffer (20 mM HEPES, pH 7.5, 500 mM NaCl, and 20 mM imidazole). Cells were lysed using the high-pressure homogenizer, and cell debris was removed by centrifugation at 4°C and 19 000 × *g* for 30 min. After centrifugation, the clear supernatant was loaded onto the pre-equilibrated Ni-NTA column (Smart-Lifesciences). Through stepwise washing, the protein was finally eluted using the elution buffer (20 mM HEPES, pH 7.5, 500 mM NaCl, and 300 mM imidazole). Eluate obtained was further purified by size-exclusion chromatography (SEC; HiLoad 16/600 Superdex 200 prep grade column, Cytiva). Finally, the protein stored in buffer containing 50 mM Tris (pH 8.0) with 100 mM NaCl were concentrated and flash-frozen in liquid nitrogen and stored at −80°C until use.

### Size-exclusion chromatography coupled with multi-angle light scattering

The oligomeric state and absolute molecular weight of Cdn1 were determined by SEC coupled with multi-angle light scattering (SEC-MALS). Briefly, protein samples were prepared at a concentration of 10 mg/ml in the sodium phosphate buffer (50 mM NaH_2_PO_4_/Na_2_HPO_4_, pH 7.4, 300 mM NaCl) and filtered through a 0.22 μm membrane prior to injection. A 10 μl sample was injected and separation was performed on TSKgel G3000SWXL column (TOSOH) equilibrated with the same buffer at 0.5 ml/min and 8°C using Waters/Arc-HPLC system at 280 nm. The eluent passed sequentially through an MALS detector (Wyatt/DAWN^®^) and differential refractive index (dRI) detector (Wyatt/Optilab^®^). Molecular weight and oligomeric state were calculated using ASTRA software (Wyatt Technology), based on light scattering and dRI signals, assuming a dn/dc of 0.185 ml/g.

### RNase and DNase activity assays

**Figure 1.**
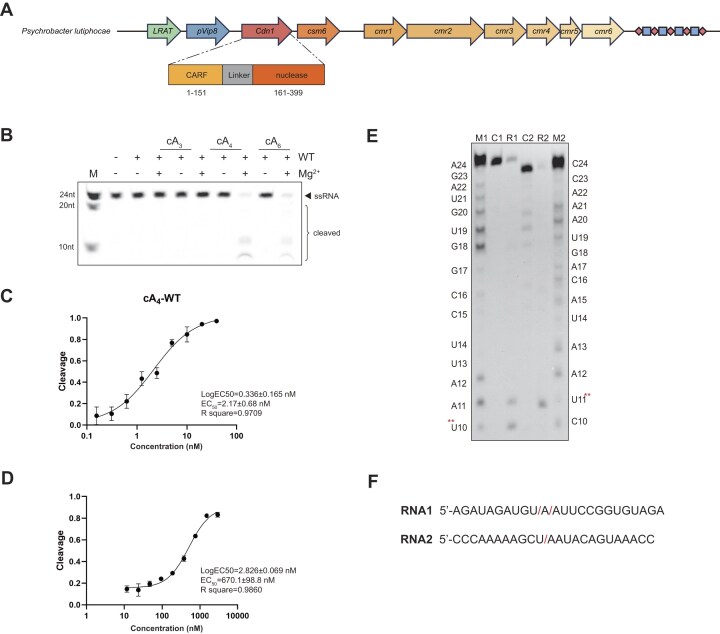
Cdn1-mediated ssRNA cleavage activity upon cA_4_ and cA_6_ activation. (**A**) Schematic representation of *Psychrobacter lutiphocae* type III-B CRISPR–Cas system. (**B**) Analysis of Cdn1-mediated cleavage of RNA1 over 30 min in the presence of cA_3_, cA_4_, or cA_6_, with or without Mg^2+^, conducted in triplicate. RNA marker (M) included 24 nt, 20 nt, and 10 nt. (**C** and **D**) Half-maximal effective concentration (EC₅₀) assay for RNA1 cleavage activated by cA_4_ and cA_6_. *X*-axis values are plotted as the inverse of the log-transformed concentrations. Data were obtained in triplicate. (**E**) Substrate preference of Cdn1, showing preferential hydrolysis of phosphodiester bonds adjacent to uridine (U) or adenosine (A) bases in ssRNA, as determined by cleavage assays using RNA1 (R1) and RNA2 (R2). For control samples 1 and 2 (C1 and C2), cA_4_ was not added. RNA markers 1 and 2 (M1 and M2) were produced as described in methods. (**F**) Sequences of RNA substrates with slashes indicating the cleaved sites. The experiment has been repeated three times.

RNA cleavage assays were conducted using 100 nM 5′ FAM-labeled RNA substrates and 50 nM Cdn1, with either 200 nM cA_4_ or 1 µM cA_6_/cA_3_ in a reaction buffer (20 mM HEPES, pH 7.5, 100 mM NaCl, and 2 mM MgCl_2_) at 37°C for 30 min. Three 5′ FAM-labeled RNAs (24 nt, 20 nt, and 10 nt) were mixed to serve as an RNA marker in Fig. 1B. EC_50_ assays were conducted with Cdn1 at 500 nM, with agonist titrations starting at 40 nM (cA_4_) and 4 µM (cA_6_), respectively, incubated for 3 min. For ssDNA cleavage assays, the concentration of Cdn1 was increased to 500 nM and the reaction time was extended to 30 min to ensure that no DNase activity was detected. Inhibitor competition assays were performed under the same conditions as the RNA cleavage assays except the reaction time was 5 min, using a 100-fold molar excess of cA_3_ relative to Cdn1. cA_3_ was either pre-incubated with Cdn1 at 4°C for 30 min or added simultaneously at reaction initiation. The reactions were quenched by the addition of 2× denaturing loading dye and heated at 95°C for 5 min. Subsequently, the reactions were analyzed using 20% urea–PAGE (20% acrylamide, 6 M urea, and 1× TBE) and scanned with a Tanon MINI SPACE 3000 system. The RNA markers 1 and 2 used in Fig. 1E were created by digesting ssRNA with nuclease P1 (NEB) at 37°C for 10 s. Digestion was halted by adding 0.1 M EDTA.

### Plasmid nuclease assay

*In vitro* nuclease assays were conducted using supercoiled pUC19 plasmid with 500 nM Cdn1 and 5 µM cA_4_ in a reaction buffer composed of 20 mM HEPES (pH 7.5), 100 mM NaCl, and 1 mM EDTA. The activities of 5 mM MgCl_2_, MnCl_2_, CaCl_2_, and ZnCl_2_ were all tested. After incubating at 37°C for 1 h, 10 mM EDTA was added to stop the reaction. Control samples included the substrate with or without metal ions, cA_4_, and protein. A linear control product was incubated with BamHI (Thermo Scientific). All samples were separated on a 1% agarose gel and scanned using a Tanon MINI SPACE 3000 system.

### Crystallization and structure determination

Crystallization conditions were screened using mosquito technology through the sitting-drop vapor-diffusion method. Cdn1 at a concentration of 15 mg/ml was incubated with cA_4_ or cA_3_ in a 1:4 molar ratio at 4°C for 30 min. Crystals of cA_4_–Cdn1 for data collection were successfully grown using the hanging-drop vapor-diffusion method under conditions of 18% PEG 8000 (w/v), 0.1 M Tris (pH 7.5), and 0.2 M MgCl_2_. Meanwhile, cA_3_–Cdn1 crystals were grown using sitting-drop vapor-diffusion in a condition containing 30% PEG 400, 0.1 M calcium acetate hydrate, and 0.1 M MES (pH 6.5). The crystals were all cryo-protected by 25% glycerol in the mother liquor and then flash-frozen in liquid nitrogen.

Diffraction data were collected at BL18U1 and BL19U1 beamlines of the Shanghai Synchrotron Radiation Facility (SSRF, shanghai, China) and processed using the data-processing program packages Porpoise_XDS and xia2 [43, 44]. Structures were solved by molecular replacement with Phaser, as part of the PHENIX package [45]. CARF domain and nuclease domain of the predicted model from AlphaFold2 were split as the searching models. Atomic model adjustment and refinement was conducted iteratively using Coot and PHENIX.refine [45, 46] (Supplementary Table S1). Figures and videos were generated using PyMOL [47] and ChimeraX [48].

### Isothermal titration calorimetry

Cdn1 or W127A mutant were diluted to 10–15 µM in the isothermal titration calorimetry (ITC) buffer composed of phosphate-buffered saline (PBS) and were titrated against 60–100 µM cA_3_/cA_4_/cA_6_ in the same buffer at 25°C by using MicroCal PEAQ-ITC system. The Q125A mutant and wild-type control were titrated against cA_3_ under identical conditions, except that the buffer contained 50 mM Tris (pH 8.0) and 500 mM NaCl. The titration sequences included a single 0.4-µl injection, followed by 19 injections of 2 µl each, with 2-min interval between injections and stirring rate of 750 rpm. Calorimetric data were analyzed and the final graphs are represented using MicroCal PEAQ-ITC analysis software.

### Plasmid challenge assay

pRS22-SepCSM (or pRS22-SepCSM-Cas10^W377A/T558A^, pRS22-SepCSM-Cas10^D590A/D591A^) and pBAD-Cdn1-Target (or pBAD-Cdn1^E308A/E310A^-Target, pBAD-Cdn1-nonTarget) were co-transformed into *E. coli* BL21 (DE3) pLysS cells [24, 31, 49–51]. Single colonies were cultured in LB medium supplemented with 100 µg/ml ampicillin, 50 µg/ml kanamycin, and 34 µg/ml chloramphenicol at 220 rpm, 37°C for 2.5 h. 0.5 mM IPTG and 0.05% l-arabinose were added to induce for 2.5 h. Two microliters of a 10-fold serial dilution was applied in duplicate to LB agar plates supplemented with 100 µg/ml ampicillin, 50 µg/ml kanamycin, and 34 µg/ml chloramphenicol. Plates were incubated at 37°C overnight. The experiment was carried out with two biological replicates and at least two experimental replicates each.

### Modeling of substrate or cA_6_ into dimeric Cdn1

The model of dimeric Cdn1 with metals activated by cA_4_ was processed and subjected to HDOCK as described before [23, 52]. The RNA substrate sequence for docking is “UUUAAA.” The structure of cA_6_ was extracted from cA_6_-Card1 (PDB: 6WXY) and modeled into CARF domain of activated dimeric Cdn1.

### Sequence analysis and construction of phylogenetic tree

Homologous sequences were searched by protein BLAST in NCBI. The resulting lists were processed [22] and analyzed through Geneious Prime. Specifically, sequence lists went through multi alignment by Clustal Omega. Sequence alignment figure was portrayed by ESPript 3.0 [53]. Phylogenetic tree was generated by neighbor joining. The final figure was portrayed by iTOL website. The alignment matrix plot was generated by ChiPlot website.

### All-atom molecular dynamics simulation

The complex system was solvated with the TIP3P water model [54], and some potassium ions were added to neutralize the system. The protein and circular RNA were modeled using the AMBER ff14SB [55] and OL15 [56] force fields, respectively. To relax the system, the energy of the system was minimized using the conjugate gradient algorithm until the convergence criterion (emtol) of 80 kJ/(mol nm) was reached. The system was heated to 298.15 K using a Berendsen thermostat, with positional restraints applied to the protein, allowing for a gradual release of constraints to prevent abrupt structural changes. Subsequently, an 80-ns NPT pre-equilibration simulation without any restraints was performed, followed by a 150-ns NPT production simulation. The pressure was regulated using Parrinello–Rahman barostat with a relaxation time (τp) of 2.0 ps and a compressibility of 4.5e−5 bar^−1^, and the temperature was maintained at 298.15 K using the V-rescale thermostat, with separate coupling groups for the protein-ligand complex (protein_lig) and the surrounding environment (envir). The van der Waals and electrostatic interactions in real space were truncated at 10 Å, and the long-range electrostatic interaction was calculated using the particle mesh Ewald method. A time step of 2 fs was used in these simulations. All simulations were performed on an NVIDIA GeForce RTX 4090 GPU, alongside a 24-core CPU, and all the analysis results were based on GROMACS [57], VMD [58], gmx_MMPBSA [59], and Dulvty [60].

## Results

### Cdn1 binds three cyclic oligoadenylates but is selectively activated by cA_4_ and cA_6_

The type III-B CRISPR gene cluster in *P. lutiphocae* encodes multiple ancillary proteins, including two CARF domain-containing effectors: a DUF1887-family CARF protein (WP_019672857.1) and the CRISPR-associated ring nuclease Csm6 (WP_019672858.1), positioned adjacent to a radical S-adenosylmethionine (SAM) enzyme and a lecithin: retinol acyltransferase (LRAT)-like hydrolase (Fig. 1A). Here, we focused on the DUF1887 family CARF protein, a well-characterized accessory effector family in type III-B CRISPR systems [22–24]. This protein consists of an N-terminal CARF domain and a C-terminal PD-(D/E)xK nuclease domain connected by a loop. Sequence alignment revealed ~20% identity with its homologs TsCard1 and StCan2 within the Can2 family (Supplementary Fig. S1A and B). Phylogenetic analysis further indicated that this protein belongs to a distinct clade, diverging from the characterized StCan2 and TsCard1 (Supplementary Fig. S1C).

To characterize this CARF domain-containing protein, we expressed and purified it as a homodimer from *E. coli* (Supplementary Fig. S2A). This oligomeric state was further validated by SEC-MALS, which demonstrated that the protein exists as a homodimer in solution (Supplementary Fig. S2B). Binding affinities of the homodimer for cyclic oligoadenylates (cA_3_, cA_4_, and cA_6_) were subsequently quantified using ITC (Supplementary Fig. S2C–E). Surprisingly, unlike all previously reported CARF domain-containing proteins, which bind to only one or two ligands, this protein binds all three ligands, with dissociation constants (Kd) of 23.0 ± 8.7 nM (cA_3_), 54.6 ± 25.1 nM (cA_4_), and 740.0 ± 205.0 nM (cA_6_) (Supplementary Fig. S2C–E). ITC measurements revealed a 1:1 molar stoichiometry between the dimer and each ligand, indicating a single binding site per dimer for these secondary messengers.

Next, activation assays revealed that cA_4_ or cA_6_ (but not cA_3_) induced ribonuclease activity against single-stranded RNA (ssRNA) (Fig. 1B), with no detectable nuclease activity toward double-stranded or single-stranded DNA (Supplementary Fig. S3A and B). Given this dual-activation profile, we designated this CARF domain containing protein as Cdn1 (CRISPR-associated dual-cOAs-activated ribonuclease 1). Cdn1-mediated ssRNA degradation required Mg^2+^, Mn^2+^, or Zn^2+^, but not Ca^2+^ (Supplementary Fig. S3C). The nuclease activity was minimally affected by pH and temperature (Supplementary Fig. S3D and E). Therefore, subsequent experiments were standardized to 37°C, pH 7.5, and Mg^2+^. Quantitative EC_50_ analysis demonstrated a greater potency of cA_4_ (2.17 ± 0.68 nM) compared to cA_6_ (670.1 ± 98.8 nM) in Cdn1 activation (Fig. 1C and D, and Supplementary Fig. S3F and G), revealing the intrinsic efficacy differences between the two agonists. To probe cleavage specificity, we designed two ssRNA substrates containing all combinations of cleavage sites. We observed that Cdn1 preferentially cleaves adjacent to uridine or adenosine residues (Fig. 1E and F).

While both cA_4_ and cA_6_ activate Cdn1 *in vitro*, cA_3_ binds without inducing activation. Notably, pre-incubation with an excess of cA_3_ did not inhibit cA_4_-mediated activation but affected that of cA_6_ (Supplementary Fig. S3H). These results indicate that while cA_3_ binds Cdn1 with high affinity, it does not act as a classical inhibitor of cA_4_-induced activation, and its effect on cA_6_-mediated activation is likely attributed to competitive binding due to higher affinity, rather than representing a physiologically relevant inhibition. Collectively, our results demonstrate that Cdn1 exhibits broad-spectrum ligand binding (cA_3_/cA_4_/cA_6_), but potent activation by cA_4_ and weak activation by cA_6_.

### cA_3_ binding stabilizes the inactive conformation of the CARF domain

To elucidate the structural basis of the dual-activation nuclease activity, we made efforts to crystalize the apo- and cA_3_/cA_4_/cA_6_ bound Cdn1. The structures of cA_3_–Cdn1 and cA_4_–Cdn1 were determined by X-ray crystallography at resolution of 2.80 and 1.83 Å, respectively (Supplementary Table S1). However, no crystals were obtained for the apo Cdn1, and cA_6_–Cdn1 crystals diffracted poorly, precluding structure determination.

**Figure 2.**
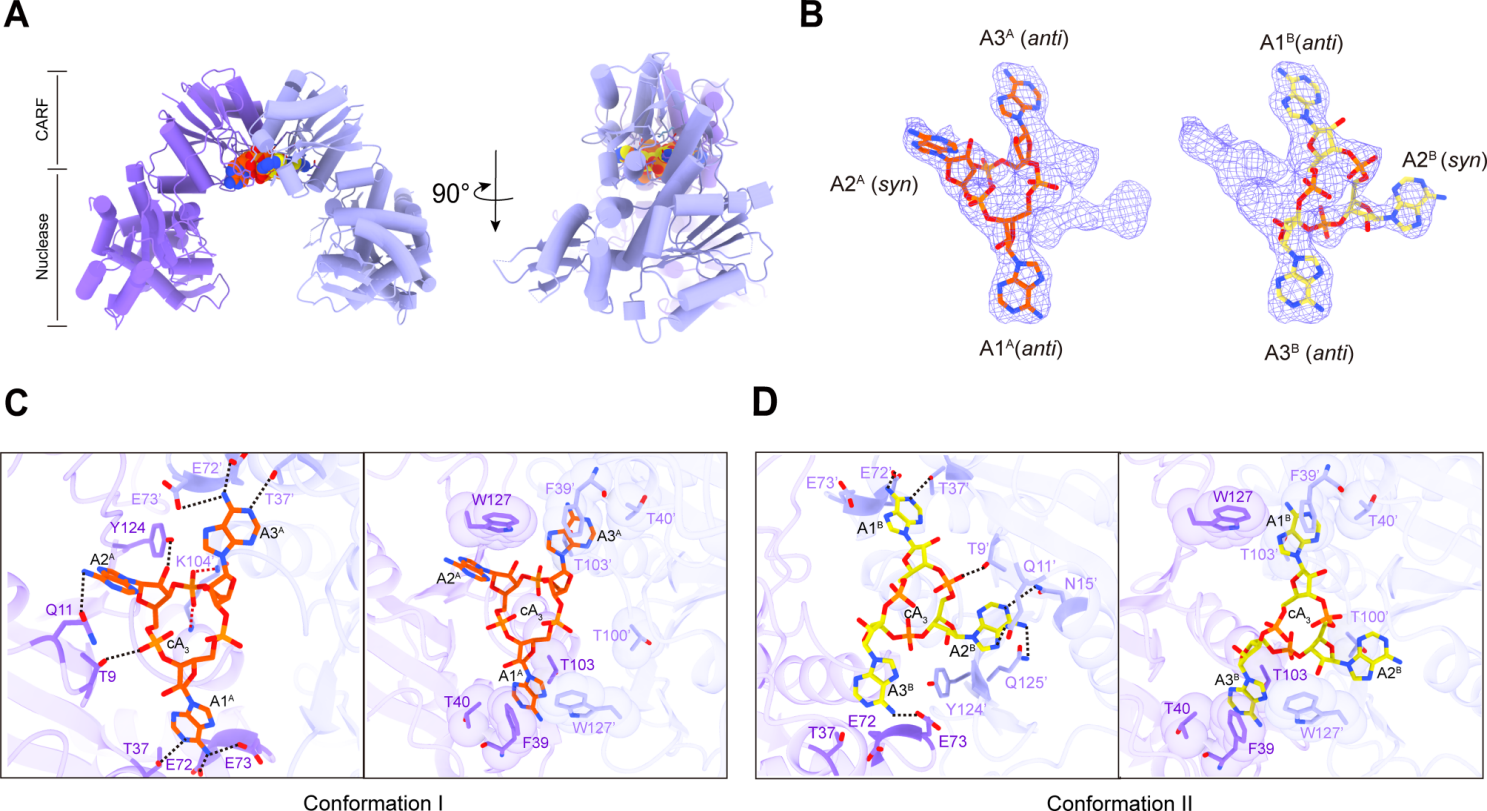
Structures of Cdn1 bound to cA_3_. (**A**) Two orthogonal views of cA_3_–Cdn1 dimer, shown in cartoon representation, showing the two subunits as distinct chains. Bound cA_3_ molecules are depicted as spheres, showing both observed conformational states. (**B**) Electron density map of cA_3_ contoured at 1.0σ (2fo–fc), highlighting two distinct conformations of cA_3_. (**C** and **D**) Intermolecular interactions between dimeric Cdn1 and bound cA_3_ of two conformations. Hydrogen bond interactions are shown on the left of each panel, while van der Waals interactions are depicted on the right. The adenylates are denoted with a superscript A in conformation I and B in conformation II.

AlphaFold2 [61, 62] predicted models were utilized for molecular replacement. The cA_3_–Cdn1 complex adopts a homodimeric architecture, with each monomer containing an N-terminal CARF domain and a C-terminal nuclease domain (Fig. 2A). Clear electron density corresponding to the cyclic oligoadenylate was observed within the central cavity of the CARF domains (Fig. 2A and B). Structural analysis revealed that the CARF domain adopts a conserved α/β Rossmann fold, while the nuclease domain features a central six-stranded β-strands core flanked by five α-helices. Unexpectedly, the cA_3_–Cdn1 complex forms a dimer interface exclusively through the CARF domains. The nuclease domains remain in an open conformation distinct from other orthologs [22, 23] (Fig. 2A). Given that such conformational opening is characteristic of the inactive state in orthologs like TsCard1 [23], we characterized cA_3_–Cdn1 as representing the inactive state supported by both structural similarity and activity assays. The CARF domain interface is mediated by specific hydrogen-bonding interactions: the side chain of E73′ interacts with S126 and S128, while the side chain of K104 forms contacts with the backbone of G102′ (Supplementary Fig. S4A, left). Crucially, we observed two conformations of cA_3_ at ~50% occupancy each within the dimer interface, representing two different binding profiles (Fig. 2B). In conformation I, A1^A^/A3^A^ are positioned within a hydrophobic pocket formed by W127/W127′, F39/F39′, and T40/T40′, and stabilized by hydrogen bonds with T37/T37′, E72/E72′, and E73/E73′ (Fig. 2C). A2^A^ forms a hydrogen bond with the side chain of Q11 and engages in a π–π interaction with W127 (Fig. 2C). Additionally, hydrogen bonds involving T9 and Y124, along with a salt bridge between the side chain of K104′ and the ribose-phosphate backbone of cA_3_, collectively stabilize the binding interface (Fig. 2C). In conformation II, A2^B^ establishes additional interactions with N15′ and Q125′, disrupting the original bonding network and eliminating the A1^B^-E73′ interaction and A3^B^-T37/E72 contacts (Fig. 2D). The slightly reduced binding affinity of the Q125A mutant is consistent with the presence of two conformations (Supplementary Fig. S4B). The dynamic rotation of adenine in A2 synergizes with its interaction with N15′ in the α1 helix, driving the coordinated movements of the α1 and α3 helices, which in turn induces a subtle rotational displacement of the C-terminal domain (Supplementary Fig. S4C and D). This conformational coupling gives rise to two distinct conformational states within the crystalline lattice, highlighting the intrinsic structural plasticity of the inactive Cdn1 architecture. Although cA_3_ fails to activate ribonuclease activity, its binding stabilizes the CARF domain, facilitating the crystallization of this inactive conformation.

### cA_4_-bound Cdn1 revealed active conformation

**Figure 3.**
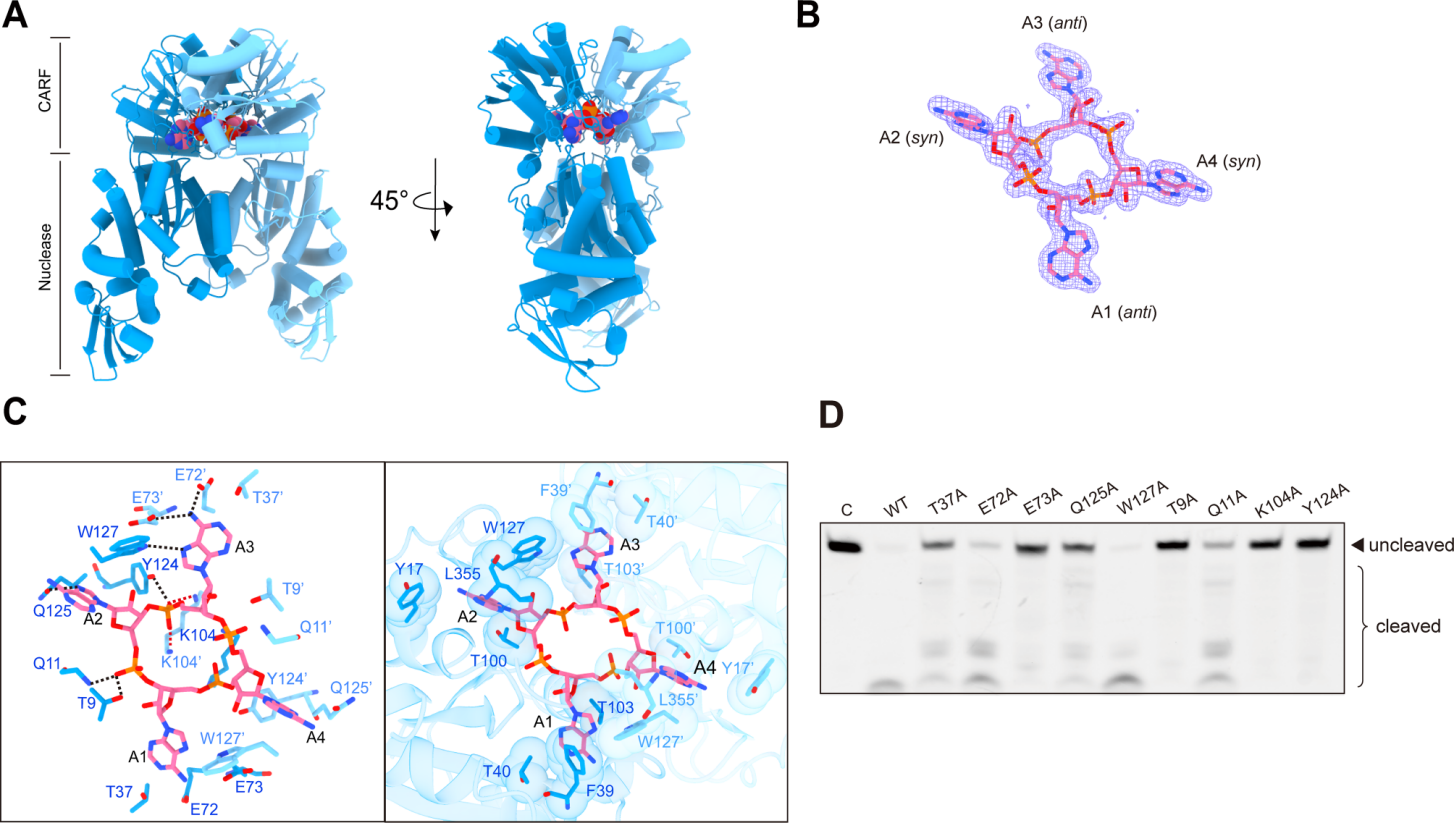
Structural basis of cA_4_ recognition and activation in Cdn1. (**A**) Two orthogonal views of the cA_4_–Cdn1 dimer in cartoon representation, showing the two subunits as distinct chains. The bound cA_4_ molecule is shown as spheres. (**B**) Electron density map for cA_4_, contoured at 1.0σ (2fo–fc). (**C**) Intermolecular interactions between cA_4_ and dimeric Cdn1. Hydrogen bond interactions are shown on the left of each panel, while van der Waals interactions are depicted on the right. (**D**) ssRNA cleavage activity of Cdn1 mutants targeting residues involved in cA_4_ binding, assessed in triplicate.

In the cA_4_–Cdn1 complex structure, cA_4_ is also positioned at the central cavity of the CARF domain (Fig. 3A and B), adopting distinct glycosidic bond conformations, with A1 and A3 in anti and A2 and A4 in syn orientations (Fig. 3B). Similar to the cA_3_ binding mode, A1/A3 is primarily recognized by T37/T37′, E72/E72′, E73/E73′, and W127′/W127 via hydrogen-bonding interactions, and are anchored within the binding pocket through hydrophobic interactions involving F39/F39′, W127′/W127, and T40/T40′ (Fig. 3C). Simultaneously, the side chains of the highly conserved residues T9, K104, and Y124 still interact with the ribose-phosphate backbone of cA_4_ (Fig. 3C). However, unlike in the cA_3_–Cdn1 structure where Q11 engages A2 via its side chain, in the cA_4_–Cdn1 complex, Q11 interacts with the ribose-phosphate backbone through its backbone amide group (Figs 2C, D, and 3C). Additionally, A2/A4 forms hydrogen bonds with Q125′/Q125 while being embedded within a hydrophobic pocket composed of Y17′/Y17, T100′/T100, and L355′/L355 (Fig. 3C). Mutagenesis studies revealed that Cdn1 variants T9A, K104A, and Y124A exhibited severely reduced cA_4_-induced nuclease activity, which correlated with disrupted ribose-phosphate backbone interactions (Fig. 3C and D). The Q11A mutant, however, retained most of its activity upon cA_4_ binding, suggesting that Q11-cA_4_ interaction is dispensable for activation relative to the essential T9 interaction (Fig. 3C and D). Activity assays revealed that residues T37, E73, and Q125 potentially mediate base interactions are important for catalytic activation, whereas E72 and W127 are less critical for cA_4_-induced activation (Fig. 3D). Notably, an interaction network involving E72, E73 and adjacent residues S128′, W127′, F39, together with A4 in cA_4_, significantly stabilizes the active conformation through hydrogen bonding and hydrophobic interactions [Supplementary Fig. S4A (right) and E].

### cA_4_-induced conformational changes facilitate the formation of an active center in the nuclease domain

**Figure 4.**
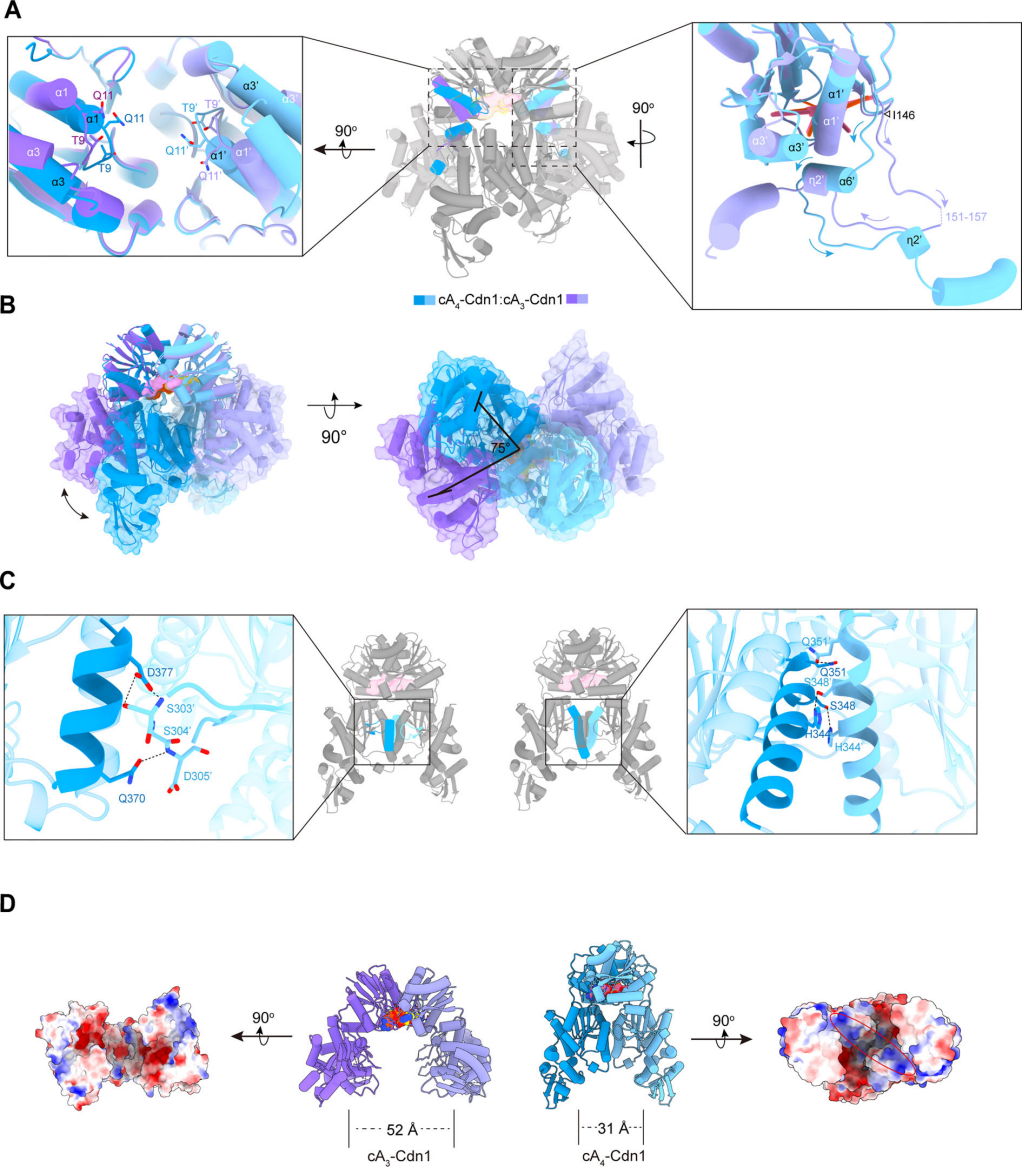
cA_4_-induced conformational changes in Cdn1 leading to activation. (**A**) Structural superposition of CARF domains in the cA_3_-bound and cA_4_-bound states. The central graph is shown as overview, while the right close-up highlights the structural rearrangement of helixes following I146 (indicated by a triangle) in the nuclease domain. (**B**) Structural alignment of cA_3_-bound and cA_4_-bound Cdn1 using CARF domains as the reference. The nuclease domains are shown as surface representations. (**C**) Dimerization interface within the nuclease domain in the active state. cA_4_ is depicted as a surface representation. Close-up views illustrate key interactions among highlighted structural motifs. (**D**) Electrostatic surface representation of the nuclease domains in the cA_3_-bound and cA_4_-bound states. Positively charged regions within the catalytic pocket are marked with ellipses. The distances between nuclease domains of two states are labeled.

To elucidate the activation mechanism, we compared the inactive and active states of Cdn1. Crucially, upon activation, we observed an inward displacement of the loop (residues 8–12), α1 (residues 13–24), and α3 (residues 45–54) toward the central pocket within the CARF domain (Fig. 4A, left). As discussed previously (Fig. 3C), the cooperative binding of T9 and Q11 to the ribose-phosphate backbone of cA_4_ induces this movement (Fig. 4A, left). These structural rearrangements propagate allosterically to the nuclease domain via the linker region (residues 151–157), ultimately facilitating catalytic activation (Fig. 4A, right).

In the inactive state, the electron density of the linker region (residues 151–157) between the CARF and nuclease domains was poorly defined, indicating intrinsic flexibility. Concurrently, residues 169–171 form an ɳ2 motif within the nuclease domain, stabilizing α1 and α3 in the CARF domain. Upon cA_4_ binding, residues 151–157 undergo a dynamic transition from an intrinsically disordered state to a well-defined α-helical conformation, displacing the ɳ2 structure observed in the inactive state and serving as a scaffold between the CARF and nuclease domains. Additionally, the ɳ2 motif undergoes an ∼180° rotational transition, ultimately facing outward (Fig. 4A, right). Consequently, the nuclease domain rotates by ∼75°, resulting in its closure in the cA_4_-bound state (Fig. 4B). These conformational changes trigger the formation of a new dimerization interface and nucleic acid binding pockets in the nuclease domain (Fig. 4C and D), and most importantly, reshape the hydrolytic active center, including the coordination of catalytic metal ions.

Specifically, homodimer stabilization is reinforced by two interfacial modules: (i) reciprocal interactions between α14 (residues 369–378, Chain A) and the loop segment (residues 302′–306′, Chain B), where the carboxyl group of the D377 forms hydrogen bonds with S303′, and the side chain of Q370 in α14 interacts with the amide group of D305′ (Fig. 4C, left); (ii) symmetrical packing of α13 (residues 335–352) through dual hydrogen bonds between His344 and S348, as well as complementary side chain interactions between Q351 and Q351′ (Fig. 4C, right). On the other hand, activation induces a contraction in the nuclease domain (from 52 to 31 Å), facilitating the formation of a positively charged substrate-binding pocket (Fig. 4D). The E308 side chain adopts distinct conformations in the inactive and active states (Supplementary Fig. S5A). In the active state, the loop segment (residues 302–306), which is essential for nuclease domain dimerization, stabilizes the adjacent E308. Conversely, in the inactive state, this segment lacks observable electron density, rendering E308 flexible (Supplementary Fig. S5A). Stabilization of E308 in the active state facilitates the assembly of an interaction network involving conserved residues—E274, D310, E323, and K325—along with three ordered water molecules (Supplementary Fig. S5B). This coordinated network effectively stabilizes the essential Mg^2+^ ion, thereby establishing an optimal environment for hydrolysis (Supplementary Fig. S5B).

Despite attempts to co-crystallize the substrate by introducing mutations in the nuclease active site, no substrate density was observed in the final refined models. Consequently, we performed molecular docking of a six-nucleotide substrate (UUUAAA) into the active site of the cA_4_-bound structure [23, 52] (Supplementary Fig. S5C). In the binding pocket, K325′ and N341 stabilize the RNA backbone. The Mg^2+^ ion coordinates water molecules to bridge E308, E310, and E323 with the C2′ hydroxyl group of U3 and the phosphate group of A4. Together with a phosphodiester salt bridge formed by K345′, these interactions facilitate phosphodiester bond cleavage via electron transfer (Supplementary Fig. S5C). As expected, mutations E308A, D310A, or E323A abolished cA_4_-induced nuclease activity in Cdn1 (Supplementary Fig. S5D), confirming their essential roles in catalysis within this enzyme family.

### Rationale behind the dual induced activation of Cdn1

Biochemical assays described above demonstrate that both cA_4_ and cA_6_ activate Cdn1 *in vitro* (Fig. 1B). To determine whether this activation mode supports antiviral function under physiological conditions, we employed a type III-A CRISPR system with Cas10 variants that produce predominantly cA_4_ or cA_6_ [50], [51]. Plasmids encoding the engineered type III-A Csm complex and Cdn1 with targeting sequence were co-transformed into *E. coli* [24, 31, 50, 51]. Negative control strains included those with Cdn1 E308A/E310A mutant, non-targeting sequence, and Cas10 cyclase variant. After induced by IPTG and l-Arabinose, strains expressing Cdn1 and targeting sequence with Cas10 producing predominantly cA_4_ or cA_6_ had fewer survivals compared with negative controls (Supplementary Fig. S6A). Thus, *in vivo* experiments indicate that both cA_4_ and cA_6_ activate Cdn1 to mediate antiviral immunity.

While the cA_4_–Cdn1 structure directly explains cA_4_-mediated activation, the structural basis by which cA_6_ stabilizes an active conformation remains unresolved. Since TsCard1 is the first characterized member which can bind cA_6_ in the CARF4 clade, we compared its structural similarity with Cdn1. TsCard1 shares 24% sequence identity with Cdn1 (Supplementary Fig. S1B). Structural superposition of cA_4_–Cdn1 and cA_4_-TsCard1 (PDB code: 6WXX) revealed a similar overall architecture in their active states, with a root-mean-square deviation (RMSD) of 2.60 Å (Supplementary Fig. S6B). Although TsCard1 was the first reported to bind both cA_4_ and cA_6_, only cA_4_ binding leads to allosteric activation [23]. Considering the unique dual activation signature of Cdn1, we conducted the sequence alignment among the family members, combined with the phylogenetic analysis (Supplementary Fig. S1C). Sequence alignment of each clade revealed that residues T9, Q11, T37, K104, and Y124, which are involved in cA_4_ binding, are highly conserved across all clades. In contrast, residues responsible for recognizing A1 and A3 vary among clades (Supplementary Fig. S6C). Specifically, E72 and E73, which are highly conserved in Clade 1 are replaced by nonconserved residues in Clade 2 and Clade 3 (Supplementary Fig. S6C–E). Additionally, the conserved F39 in Clade 1, which stabilizes A1 and A3, is replaced by nonconserved residues in Clade 2 or Clade 3 (Supplementary Fig. S6C). These evolutionary substitutions result in a more enclosed cA_4_-binding pocket in cA_4_–Cdn1 compared to cA_4_–Card1 and cA_4_–Can2 (Supplementary Fig. S6F).

**Figure 5.**
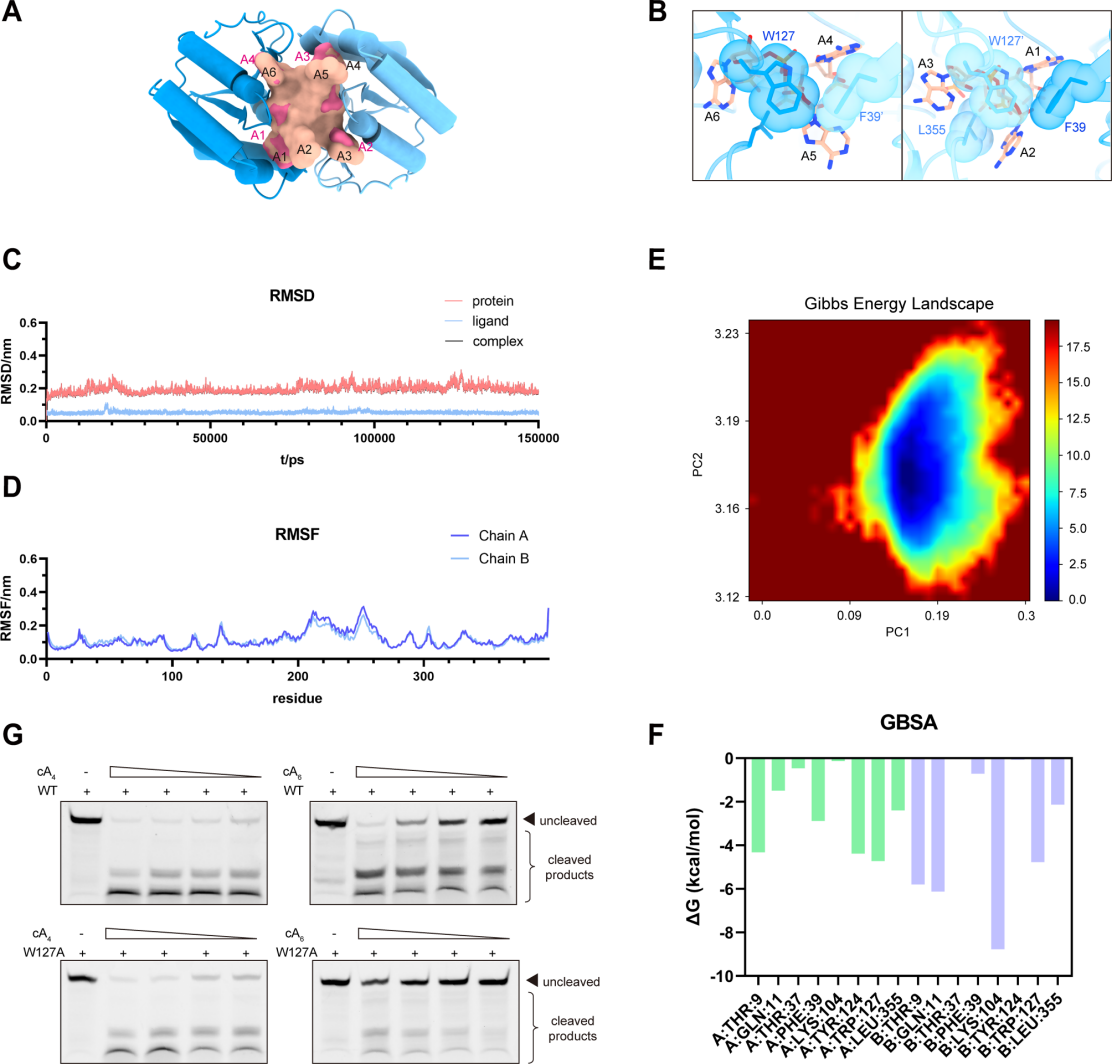
Modeled binding profile of cA_6_ in the active state of Cdn1. (**A**) Docking model of cA_6_ in the active Cdn1 structure. The adenines A1, A3, A4, and A6 of cA_6_ align with the adenines A1, A2, A3, and A4 of cA_4_, respectively. cA_6_ is shown in salmon. (**B**) The interaction profile of A2 and A5 bases with F39, W127, and L355 in cA_6_−Cdn1 model. (**C**) RMSD of cA_6_–Cdn1 complex model for 150 ns MD simulation. The interaction fraction profile during MD simulation. (**D**) RMSF of both protein chains and ligand chain in the MD simulation. (**E**) Gibbs energy landscape generated using Dulvty for cA_6_–Cdn1. (**F**) Estimation of the binding free energy of the complex based on the GBSA method, performed using the gmx_MMPBSA script with a sampling interval of 30 000 frames. (**G**) Effects of W127A mutation on cA_4_- and cA_6_-induced activation of Cdn1.

Apart from the main difference discussed above, comparative analysis of the cA_4_-binding pockets revealed shared conserved residue interactions between TsCard1 and Cdn1 (Supplementary Fig. S7A). Given that the bases (A1, A2, A4, and A5) of cA_6_ in the cA_6_-TsCard1 structure (PDB code: 6WXY) occupy similarly positions to those in the cA_4_–Card1 structure (PDB code: 6WXX) [23], we substituted cA_4_ in the cA_4_–Cdn1 structure with cA_6_ from the cA_6_–Card1 complex to investigate how cA_6_ binding induces Cdn1 activation (Fig. 5A). Unlike in cA_6_–TsCard1 structure, where the A2 and A5 bases of cA_6_ push away L339, preventing the conformational rearrangement required for activation [23], the cA_6_–Cdn1 model accommodates these bases without steric clashes, thereby enabling the conformational changes necessary for activation (Supplementary Fig. S7B). The A2 and A5 bases are stabilized through hydrophobic contacts with W127 and L355 (Fig. 5B). Molecular dynamics (MD) simulations were performed to assess the dynamic stability of the simulated cA_6_–Cdn1 binding mode. MD simulations over a 150 ns confirmed the structural stability of the cA_6_–Cdn1 complex, with the RMSD plateauing at an average of 0.2–0.3 nm (Fig. 5C). Both the CARF and nuclease domains exhibited low structural flexibility, with root mean square fluctuation (RMSF) values mostly below 0.2 nm (Fig. 5D), and the trajectory analysis indicated the sustained stability of the dimerization interface throughout the simulation (Supplementary Video S1). Collectively, these findings suggest that both domains remain structurally stable in the activated state upon cA_6_ binding, whereas linker region plasticity may contribute to slight fluctuations. Furthermore, MD-based principal component analysis confirmed that cA_6_ stabilizes the complex in a bound state (Fig. 5E). Binding free energy calculations indicated that the key conserved residues T9, Q11, T37, K104, and Y124 made significant favorable (negative) contributions as expected, despite the asymmetric distribution (Fig. 5F and Supplementary Fig. S7C). Apart from these conserved positions, residues F39, L355, and especially W127, also displayed significant negative energy contributions, suggesting an additional stabilizing role in the docking cA_6_–Cdn1 complex (Fig. 5F and Supplementary Fig. S7C). The similar MD simulation was also conducted for the experimentally observed cA_4_–Cdn1 structure, which likewise demonstrated a stable binding conformation during the simulation (Supplementary Fig. S7D–F and Supplementary Video S2). Together, these simulations support the plausibility of the proposed dual-induced activation mode. Furthermore, enzyme activity assays showed that the W127A mutation selectively impaired cA_6_-induced activation, while cA_4_-mediated activity remained comparable to wild-type Cdn1 (Fig. 5G and Supplementary Fig. S7G), supporting the role of W127 as a structural gate stabilizing cA_6_ binding. This is consistent with the reduced cA_6_ affinity observed in ITC assays of the W127A mutant (Supplementary Fig. S7H). Superimposition of the CARF domain monomers between the active states of Cdn1 and TsCard1 revealed a slight rotation in the opposing chain of Cdn1, particularly around the activation-critical loop and α1 helix (residues 13–24) (Supplementary Fig. S7I). This rotation likely arises from the dimer interface formed by the additional highly conserved E72–E73 pair characteristic of the clade to which Cdn1 belongs (Supplementary Fig. S6D and E), resulting in a more staggered active homodimer conformation. Such an arrangement provides greater spatial accommodation for the A2/A5 bases, as discussed above (Supplementary Fig. S7B). Collectively, these findings demonstrate that cA_6_ binding induces the conformational rearrangements necessary for Cdn1 activation and provide direct evidence supporting the intrinsic dual activation mechanism of Cdn1.

## Discussion

Here, we define Cdn1 as a dual-activated nuclease from the Can2 family associated with the type III-B CRISPR–Cas system. Cdn1 uniquely binds three types of cOAs, adding a significant layer of complexity to the understanding of CRISPR-associated accessory proteins.

The cA_3_–Cdn1 complex represents the first resolved structure of a cA_3_-bound CARF domain. NucC, a well-characterized cA_3_-activated effector, was initially identified in CBASS systems [41, 42, 63]. Binding of cA_3_ to its conserved allosteric pocket induces the closure of three hairpin loops, effectively enclosing the cA_3_ molecule [42, 63]. This conformational change drives the transition from trimeric subunits to a homohexameric assembly, enabling dsDNA cleavage [42]. Recent studies have also revealed cA_3_-mediated activation of TIR-SAVED effectors, wherein cA_3_-induced helical assembly properly aligns TIR domains to facilitate NAD^+^ hydrolysis [30, 31]. cA_3_ binds at the interface between adjacent SAVED domains, inducing head-to-tail multimerization of the entire effector—a characteristic feature of SAVED domain-fused effectors. While NucC employs ternary symmetric binding and TIR-SAVED utilizes sandwich-like multimerization, Cdn1 exhibits a distinct mode of cA_3_ coordination, underscoring the diversity of cA_3_ recognition strategies among cyclic oligonucleotide signaling systems. While the physiological significance of this high-affinity cA_3_ binding remains to be further explored, these findings provide new insights into the structural and functional versatility of cyclic nucleotide signaling systems.

Our structural analysis reveals that cA_4_ binding drives Cdn1 activation through coordinated domain reorganization. Specifically, cooperative interactions between T9 and Q11 and the ribose-phosphate backbone of cA_4_, pull the loop segment, inducing an inward displacement of the α1 and α3 helices. This remodeling culminates in a 75° counterclockwise rotation of the nuclease domain, promoting dimerization and facilitating the formation of substrate-binding pockets with reconfigured catalytic sites (Supplementary Video S3). While these rearrangements partially align with the established “pull and rotate” activation mechanism described for other CARF-fused proteins [22–24], this pronounced conformational change and *de novo* formation of dimer interfaces, distinguishes Cdn1 from previously characterized CARF-fused effectors within this family.

To elucidate the specificity of cA_6_-mediated activation, we constructed a cA_6_–Cdn1 model using the cA_6_–TsCard1 structure (PDB: 6WXY) as a template. This model rationalizes why cA_6_ activates Cdn1 but not TsCard1, providing key mechanistic insights despite the absence of an experimentally resolved cA_6_-Cdn1 structure. Molecular dynamics simulation further supported the structural convergence and stability of the cA_6_–Cdn1 complex model. However, alternative binding configurations or dynamic interaction modes may exist that are not captured within the simulated timescale, and the inferred energetic contributions are subject to force-field and sampling limitations. Given these considerations, we further explored whether independent evolutionary signatures provide additional support for the proposed dual-activation mechanism. Sequence alignment identified the characteristic E72–E73 residue pair unique to the Cdn1 clade. Structural comparisons suggest that this conserved pair contributes to its dual activation mechanism. Moreover, plasmid challenge experiments provide complementary *in vivo* evidence supporting the functional relevance of dual activation. These data bridge the computational model with cellular function and reinforce the proposed dual-activator framework. Taken together, by elucidating the distinct mechanisms of cA_4_ and cA_6_ activation, we reveal for the first time a class of CARF nucleases, exemplified by Cdn1, that employ a dual activation mechanism.

In conclusion, Cdn1 represents a unique class of potential dual-activated nucleases that expand the functional diversity of CRISPR-associated CARF effectors. Its capacity for differential activation by cA_4_ and cA_6_ highlights the adaptability to amplify immune responses in coordination with type III CRISPR–Cas complexes, enhancing anti-invasive nucleic acid defense. Notably, Cdn1’s ability to bind multiple cyclic oligonucleotides (cA_3_, cA_4_, and cA_6_) reflects an evolutionary specialization within the Can2 family (Supplementary Figs S1C and S6C-D). We propose that this structural plasticity serves like a strategy to maximize defensive robustness during horizontal gene transfer and microbial competition, which requires rapid integration into host regulatory networks while maintaining broad-spectrum anti-invasive efficacy. Such evolutionary innovation ensures sustained protection against diverse genetic invaders while maintaining compatibility with CRISPR–Cas regulatory networks.

## Supplementary Material

gkaf1524_Supplemental_Files

## Data Availability

The atomic coordinates have been deposited into Protein Data Bank with PDB 8Z4I (cA_3_–Cdn1) and 9U49 (cA_4_–Cdn1).
