# Supplementary material for: Structural plasticity enables broad cAn binding and dual activation of CRISPR-associated ribonuclease Cdn1
Source: Nucleic Acids Res. 2026 Jan 22;54(3):gkaf1524. doi: 10.1093/nar/gkaf1524 (PMC12825302; doi:10.1093/nar/gkaf1524)
Supplement: gkaf1524_Supplemental_Files [file gkaf1524_supplemental_files.zip › 060126025924_Supplementary_Information.pdf]

Supplementary Information

Structural Plasticity Enables Broad cAn Binding and Dual Activation of CRISPR-associated Ribonuclease Cdn1

Wenxuan Zhang[1], Jianping Kong[1], Yuqin Zeng[1], Yunning Su[2], Sijun Zhang[2, 3], Yutao Li[1], Chunyi Hu[4, 5], Qihua Chen[6, 7*], Yibei Xiao[1, 8*], Meiling Lu[2, 3*]

[1] State Key Laboratory of Natural Medicines, School of Pharmacy, China Pharmaceutical University, Nanjing 211198, China,
[2] Department of Biochemistry, School of Life Science and Technology, China Pharmaceutical University, Nanjing 211198, China
[3] Jiangsu Provincial Key Laboratory of Biology and Therapeutics for Immune System-Related Diseases, China Pharmaceutical University, Nanjing 211198, China
[4] Department of Biological Sciences, Faculty of Science, National University of Singapore, Singapore 117543, Singapore
[5] Precision Medicine Translational Research Programme (TRP), Department of Biochemistry, Yong Loo Lin School of Medicine, National University of Singapore, Singapore 117543, Singapore
[6] Department of Neurology, the Second Xiangya Hospital, Central South University, Changsha 410008, China
[7] Clinical Medical Research Center for Stroke Prevention and Treatment of Hunan Province, Department of Neurology, the Second Xiangya Hospital, Central South University, Changsha 410008, China
[8] Chongqing Innovation Institute of China Pharmaceutical University, Chongqing 401135, China

* To whom correspondence should be addressed. Tel: +86 25 86185335; Email: lumeiling@cpu.edu.cn

Correspondence may also be addressed to Yibei Xiao. Email: yibei.xiao@cpu.edu.cn; Qihua Chen. Email: chenqihua1984@163.com

Supplementary Table 1-2

Supplementary Figure 1-7

Supplementary Video 1-3

Supplementary Table 1. Crystallographic data collection and refinement statistics

	**cA_4-Cdn1**	**cA_3-Cdn1**
**PDB code**	9U49	8Z4I
**Data collection**		
Wavelength	0.979 Å	0.979 Å
Resolution range	44.15-1.78 (1.83-1.78)*	45.33-2.79 (2.86-2.79)*
Space group	P 2_1 2_1 2	C 1 2 1
Unit cell		
a, b, c (Å)	89.93, 132.44, 75.9	152.49, 61.30, 103.57
α, β, γ (°)	90, 90, 90	90, 97.55, 90
Total reflections	916535 (81718)	157954 (11888)
Unique reflections	72017 (7071)	23893 (1748)
Multiplicity	12.7 (11.6)	6.6 (6.8)
Completeness (%)	99.8 (99.6)	99.9 (99.9)
Mean I/sigma(I)	18.5 (5.1)	12.3 (1.2)
R-merge	0.109 (1.58)	0.132 (1.80)
R-meas	0.114 (1.65)	0.143 (1.94)
R-pim	0.032 (0.487)	0.055 (0.742)
CC1/2	0.997 (0.873)	0.998 (0.462)
**Refinement**		
Resolution range (Å)	32.93-1.9	45.33-2.8
R-work/ R-free	0.1890/0.2265	0.2476/0.2777
Number of atoms		
macromolecules	6494	6418
ligands	133	132
solvent	809	33
RMS (bonds, Å)	0.007	0.003
RMS (angles, °)	0.86	0.56
Ramachandran favored (%)	97.61	96.66
Ramachandran allowed (%)	2.39	3.34
Ramachandran outliers (%)	0.00	0.00

*Values in parentheses are for highest-resolution shell. One crystal was used to solve each structure.

Supplementary Table 2. Synthetic nucleic acid sequences

Oligonucleotides	Sequence 5’-3’	Note
plCdn1-6×His	atgaaaacactatttgtaactgctacgggacaaacggaggcgaactactacaccatttggcacctgttccgcagccagaccaatattgaaaagatcgttgttttaagcaccgatttcacccgtaaaaagaacctgttgtccaatttgatggaactgctcaacttgttagacaccggtatccacgtggaagaacttcacctaccggatggtattgaggagaagtccatttcggacatcaaggcggttatttatcagtggattgacaacaaccagccgaaagagatcatttttaacgtgacgggcggtacaaaactgatttctttcgcccaagatcagatcgctgcgaataacccgaattacagctgtgtttatcaatcatggtccaataaccagctggtttggtataacaccccggataaaccgctggaagacatcatcctgcctgaaaacatcgcggtgcgcctgaaaggtcatggttacgaccaaattagtagcgaaactgcgtttttggatctgccgattgagcagtaccattacatcgcccaactctataaactgattaagatcgacttcaccaaggcgcaacgtctggtcagctacctgaactacctggtcagctctttcgatcaaaaggcggtttcctatccgtactgctttgagatcaaaaaggagggcagcttcctgtctcttgccggctggatcaagaccctggcacaggcagcgaaaccatttatccagttggagagcctggacgaccagaagagcaagattaccttcatgagcaaagaggcggctgaatttattggtggtaaatggtttgaggtgctggtgggttttctgatcactgcgtattaccaaaagaagcagacgctggtcaatatccaaatcggcctgaccttcgctaaatctagcgacggcaacgaaattgatgttgcgtacttgcttaagggccacttctattggatggagtgcaaaaccgtgaattggctgaagaaaaatgcaccgaccaccgaagttaataacaacctgcacaaactgtcctcgatttcacagggtgcaggcttgaacagccataaattctttgttagcctgtatgatatctcggaacagagccgtaaggtggctgaggacctgggcgtaatcgtgattgcgggtacggatctgttcaaattcgaccgtttttaggtgaagtggctcaccaccaccaccaccactga	For protein expression and purification
Target sequence	acgtatgccgaagtatataaatcatcagtacaaag	For plasmid challenge assays
T9A primer F	ctgctgctggacaaacggaggcgaactactac	Mutation
T9A primer R	tttgtccagcagcagttacaaatagtgttttcatggtatatctcct	
Q11A primer F	tacgggagctacggaggcgaactactacaccatt	
Q11A primer R	tccgtagctcccgtagcagttacaaatagtgttttcatggt	
T37A primer F	ttaagcgctgatttcacccgtaaaaagaacctgttgtcc	
T37A primer R	gaaatcagcgcttaaaacaacgatcttttcaatattggtctggct	
E72A primer F	ggtattgctgagaagtccatttcggacatcaaggc	
E72A primer R	gacttctcagcaataccatccggtaggtgaagttcttcc	
E73A primer F	attgaggctaagtccatttcggacatcaaggcg	
E73A primer R	ggacttagcctcaataccatccggtaggtgaagttc	
K104A primer F	cggtacagctctgatttctttcgcccaagatcagatcg	
K104A primer R	atcagagctgtaccgcccgtcacgttaaaaat	
Y124A primer F	gtgttgctcaatcatggtccaataaccagctggtttg	

Y124A primer R	atgattgagcaacacagctgtaattcgggttattcgca	Mutation
W127A primer F	caatcagcttccaataaccagctggtttggtataacacc	
W127A primer R	attggaagctgattgataaacacagctgtaattcgggtt	
E274A primer F	aaatggtttgctgtgctggtgggttttctgatcact	
E274A primer R	cacagcaaaccatttaccaccaataaattcagccgc	
E308A primer F	ggcaacgctattgatgttgcgtacttgcttaaggg	
E308A primer R	atcaatagcgttgccgtcgctagatttagcgaa	
D310A primer F	aacgaaattgctgttgcgtacttgcttaagggccac	
D310A primer R	aacagcaatttcgttgccgtcgctagatttagc	
E323A primer F	ggatggcttgcaaaaccgtgaattggctgaag	
E323A primer R	ttttgcaagccatccaatagaagtggccccttaagcaa	
W377A primer F	actattGCgcgtaatgttagcagcaaactttctgatattaaagc	
W377A primer R	attacgcGCaatagttctataattaccacttgtattcattaaatcatcgccac	
T558A primer F	tcaagaGcagctacattgtctcgtcaattatcattattttttaaatacg	
T558A primer R	tgtagctgCtcttgaaattgaattatatttttctggaattccagatataaatgtagc	
D590-591A primer F	ggtgCcgCtttatttttaatcggtgcatgggatgacattatagaag	
D590-591A primer R	aaataaaGcgGcaccgcctgaatatatagcagtaatttgataattttctaataaatg	
RNA1	AGAUAGAUGUAAUUCCGGUGUAGA	5' FAM labeled for RNA cleavage assay
RNA2	CCCAAAAAGCUAAUACAGUAAACC	5' FAM labeled for RNA cleavage assay
RNA-20nt	AGAUAGAUGUAAUUCCGGUG	5' FAM labeled for RNA marker
RNA-10nt	AGAUAGAUGU	5' FAM labeled for RNA marker

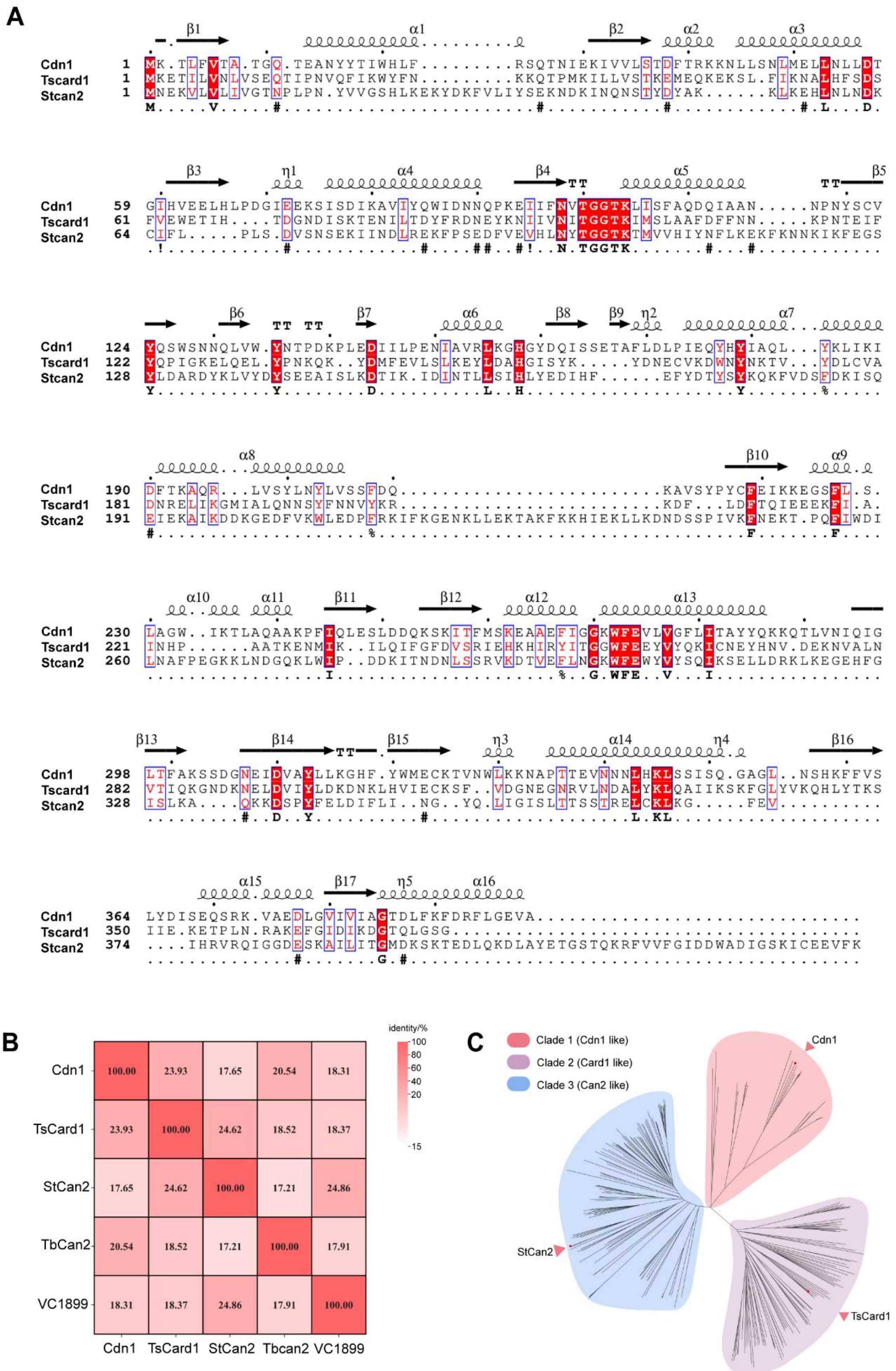
A
Cdn1
Tscard1
Stcan2
B
identity/%
Cdn1
TsCard1
StCan2
TbCan2
VC1899
100.00 23.93 17.65 20.54 18.31
23.93 100.00 24.62 18.52 18.37
17.65 24.62 100.00 17.21 24.86
20.54 18.52 17.21 100.00 17.91
18.31 18.37 24.86 17.91 100.00
Cdn1 TsCard1 StCan2 Tbcan2 VC1899
C
Clade 1 (Cdn1 like)
Clade 2 (Card1 like)
Clade 3 (Can2 like)
Cdn1
StCan2
TsCard1

Supplementary Figure 1. Structural-based sequence alignment and phylogenetic tree analysis of Cdn1 and other DUF1887 family proteins

(**A**) The amino acid sequences of Cdn1 from *Psychrobacter lutiphocae*, Card1 from *Treponema succinifaciens* (TsCard1)*,* and Can2 from *Sulfobacillus thermosulfidooxidans* (StCan2) were aligned using Clustal Omega. The alignment was visualized by ESPript 3.0. (**B**) Structural and sequence alignment matrix plot of Cdn1 and other characterized DUF1887 family proteins, generated using Swiss-Model. (**C**) Phylogenetic tree depicting the clustering of Cdn1 with other DUF1887 family proteins. Specific branches corresponding to Cdn1, TsCard1 and StCan2 are highlighted.

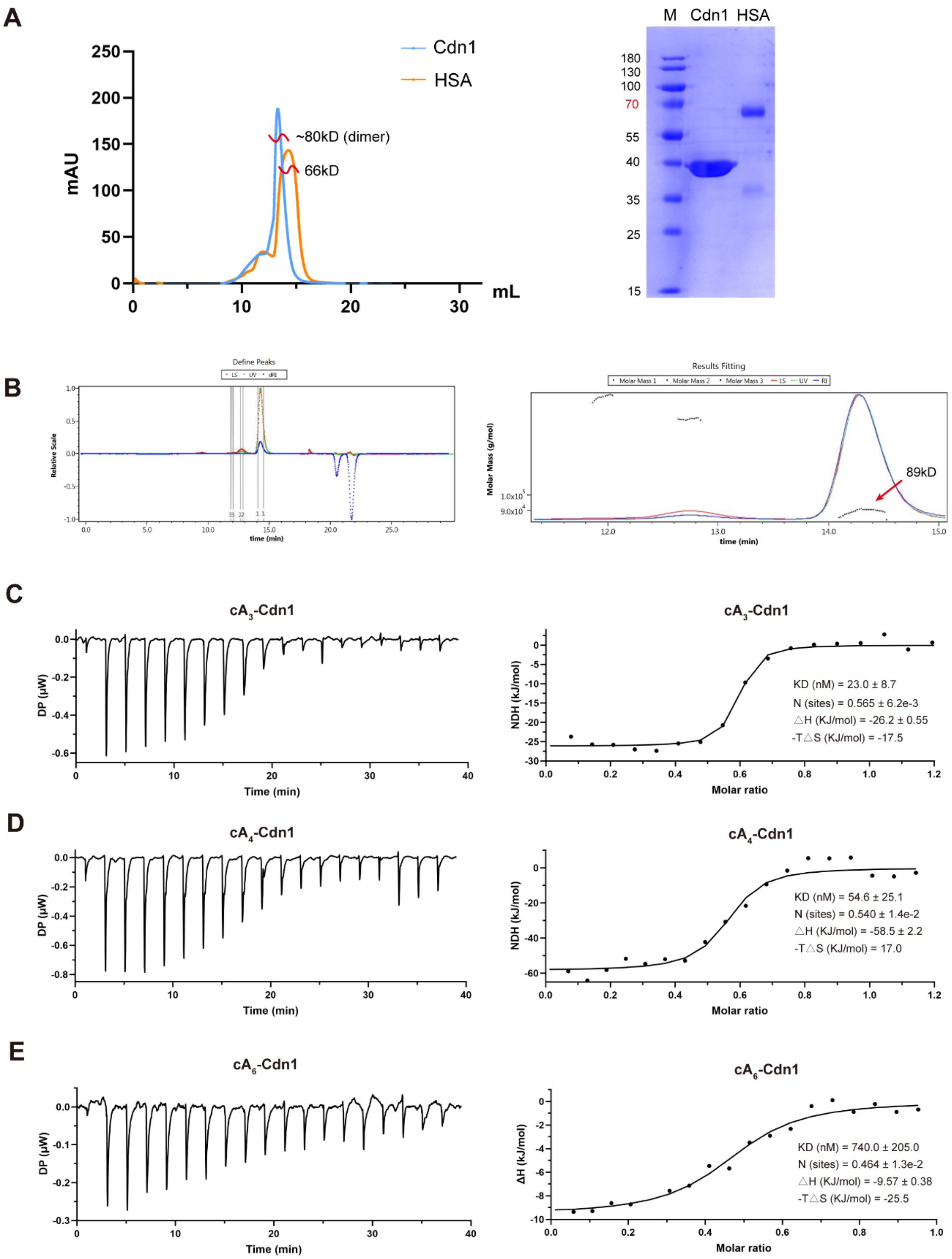

Supplementary Figure 2. ITC and SEC analyses indicate the affinity of cAn for dimeric Cdn1

(**A**) Size-exclusion chromatography (SEC) analysis of Cdn1 and human serum albumin (HSA) suggests that apo-Cdn1 exists as a dimer. (**B**) SEC-MALS analysis of Cdn1. The molar mass of

peak1 is marked on the right panel. LS, UV, and dRI represent light scattering, UV absorbance at 280 nm, and differential refractive index signals, respectively. (**C-E**) Isothermal titration calorimetry (ITC) binding curves for cA_3, cA_4, and cA_6 interacting with apo-Cdn1.

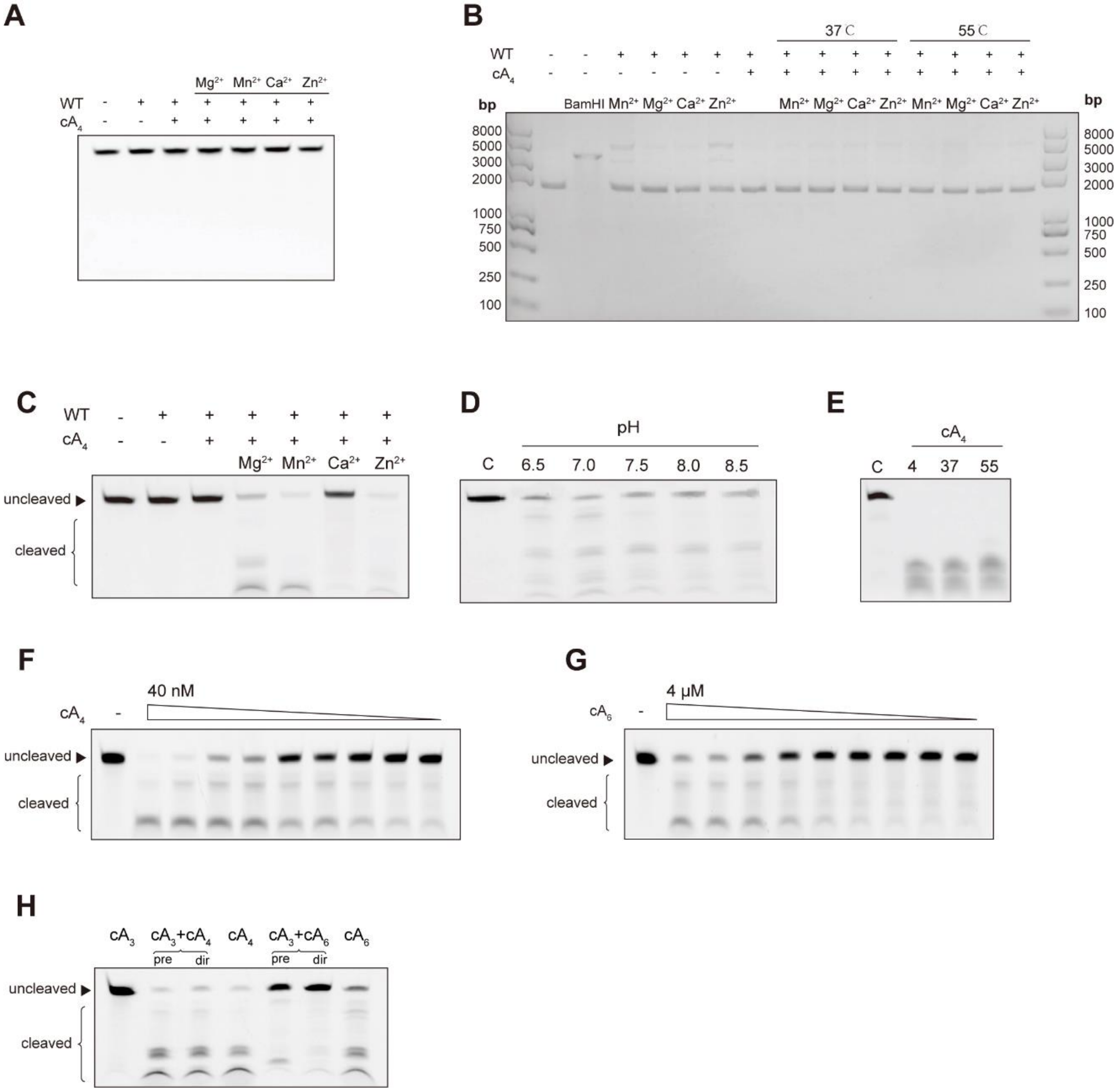

Supplementary Figure 3. Comparison of factors affecting cA_4/cA_6-induced activation

(**A**) No detectable nuclease activity in the ssDNA cleavage assay. (**B**) No detectable nuclease activity is the plasmid cleavage assay. (**C-E**) Effects of metal ions (C), pH (D) and temperature (E) on ssRNA cleavage, using RNA1 as the substrate. (**F-G**) EC50 assays for cA_4 and cA_6. The grayscale values of uncleaved bands were quantified and fitted against the concentration of cA_4 and cA_6. (**H**) Inhibitor competition assays for cA_3 versus cA_4 or cA_6. cA_3 was pre-incubated (pre) with Cdn1 or directly added at reaction initiation (dir). All experiments were performed in triplicate.

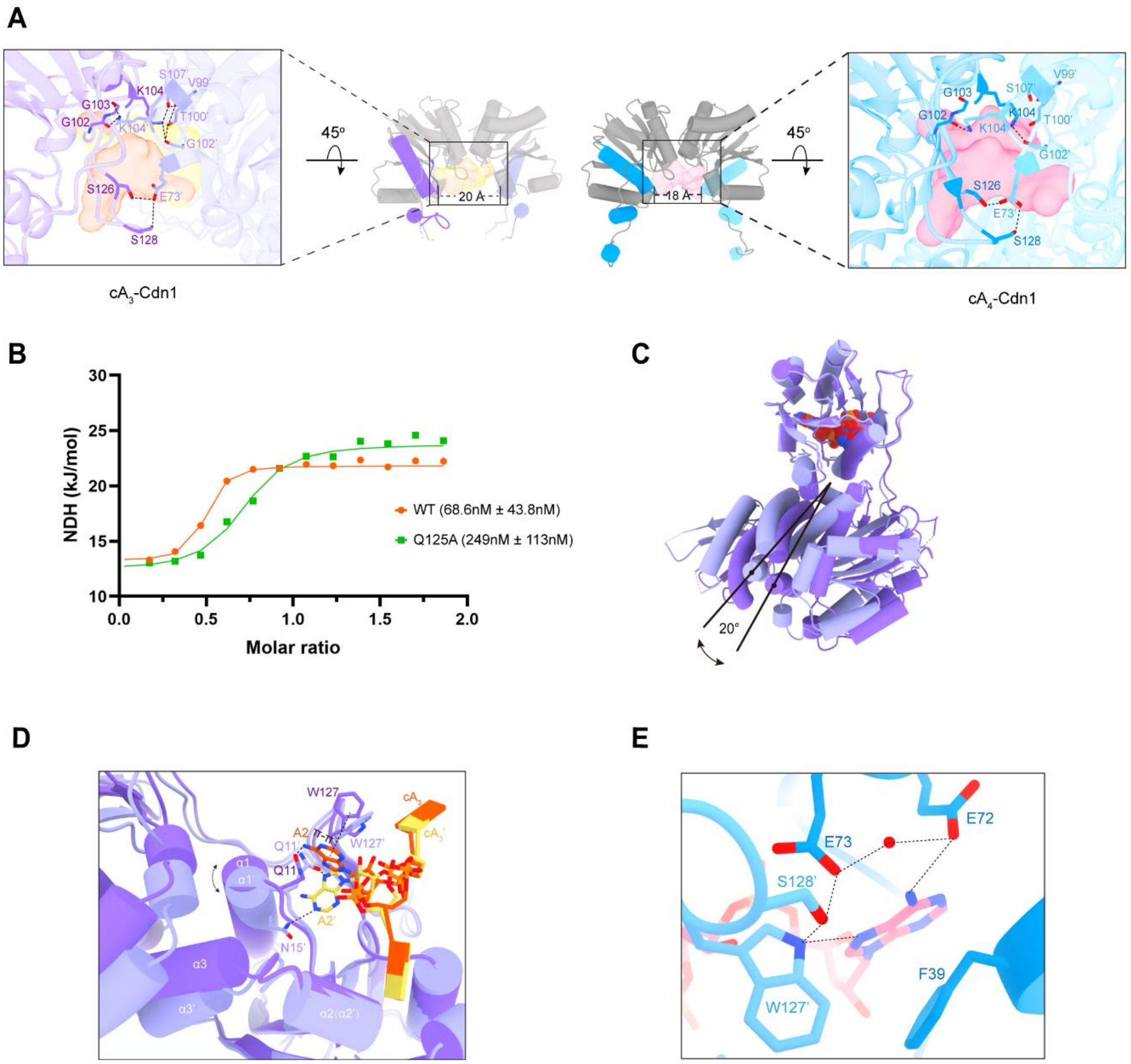

Supplementary Figure 4. Structural comparison revealing conformational changes

(**A**) Dimerization interface within CARF domain in inactive and active states. cA_3 and cA_4 are shown as surfaces, colored consistently with Fig. 2A and Fig. 3A. Close-up views highlight dimer interface interactions within the CARF domain in both states. (**B**) ITC results of wild-type Cdn1 and Q125A mutant titrated by cA_3 demonstrating the presence of two conformations of cA_3. (**C**) Superposition of two monomers from the cA_3-Cdn1 structure, aligned via the CARF domain. One monomer exhibits a ~20° rotation in the nuclease domain relative to the other. (**D**) Structural alignment of two cA_3 conformations to compare flexibility and intradomain interactions within the CARF domain. (**E**) Interaction network of E72 and E73, illustrating their structural roles.

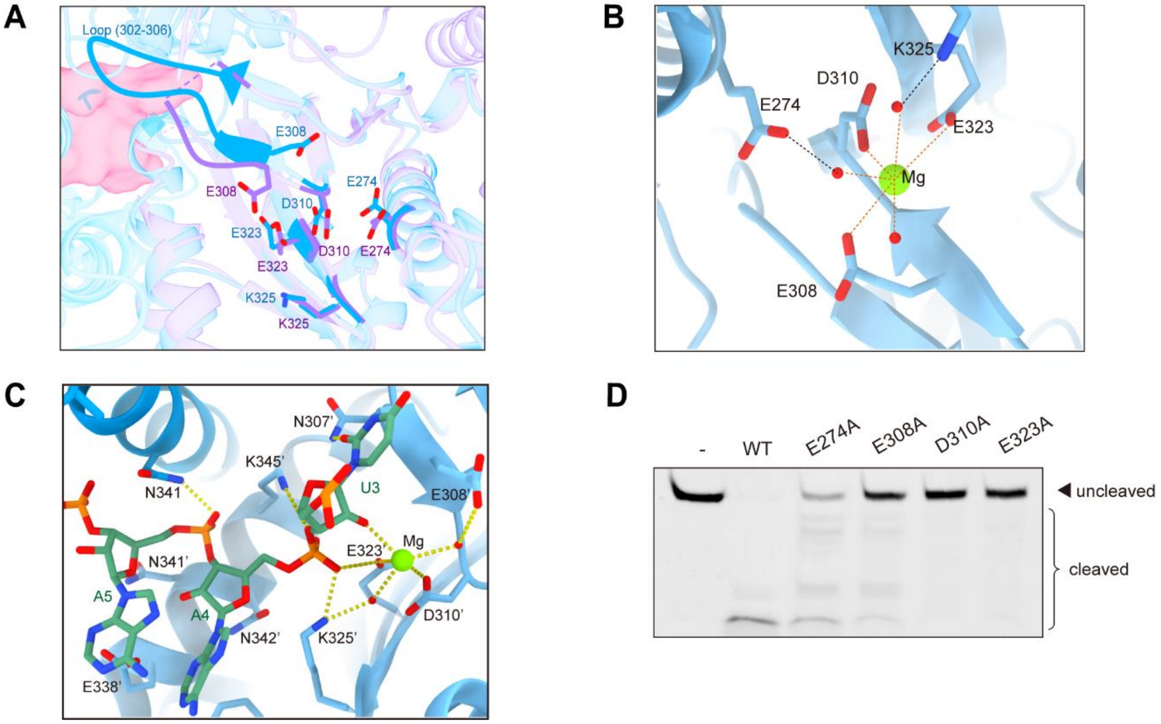

Supplementary Figure 5. Reconstruction of catalytic pocket upon activation

(**A**) Superimposition of the catalytic pocket in the nuclease domain between inactive (purple) and active (blue) states. The side chains of conserved residues are shown and labeled. (**B**) Amino acids residues lining the catalytic pocket of the cA_4-Cdn1 complex. The bound magnesium ion is shown as a green sphere, while water molecules are represented as red spheres. (**C**) Close-up view of the RNase active site with a docked RNA substrate ("UUUAAA") in the catalytic pocket. Interaction sites are indicated by yellow dashed lines. (**D**) Effects of conserved catalytic pocket residues on cA_4-induced activation.

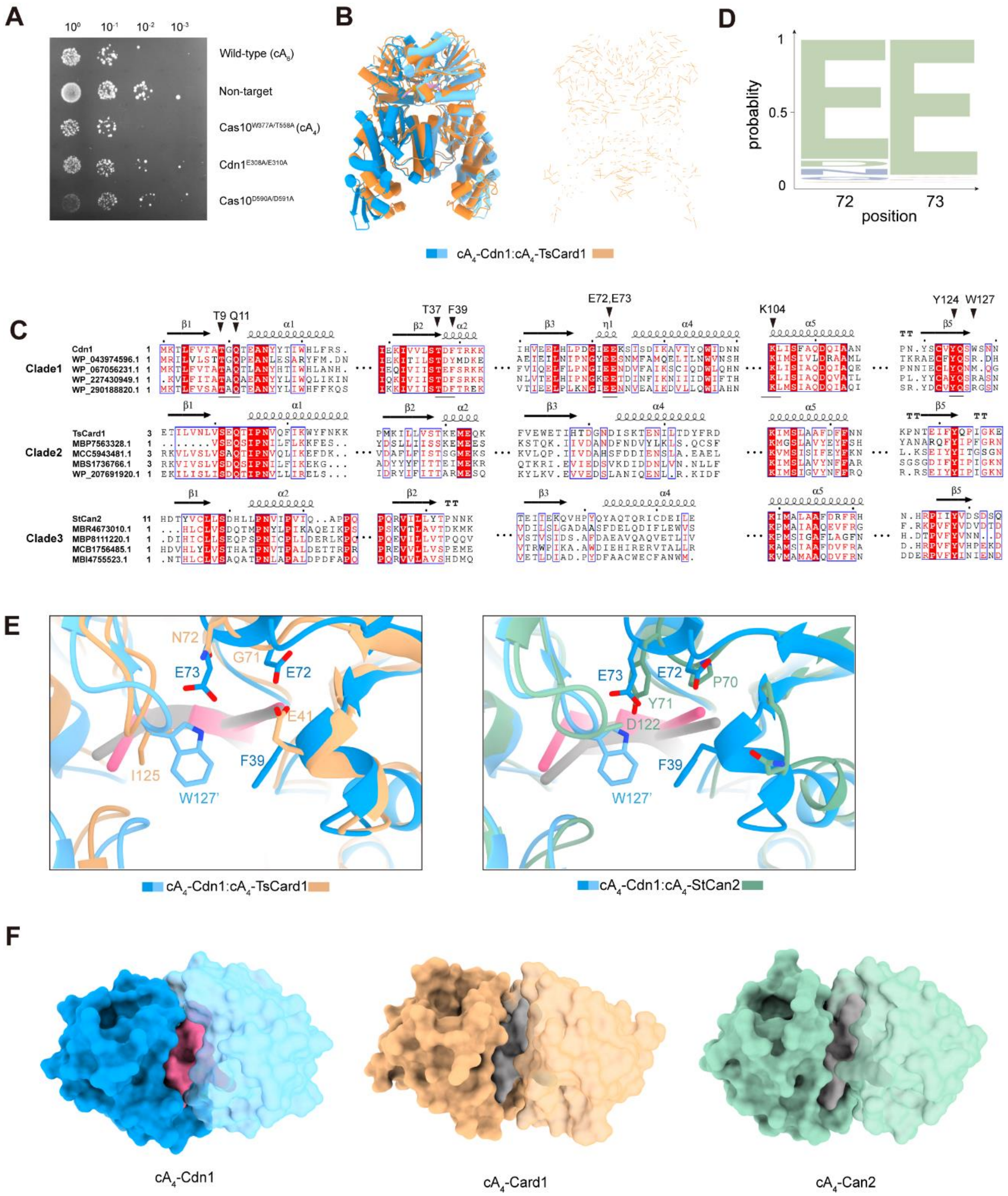

Supplementary Figure 6. Structural comparison and sequence alignment among Cdn1 and other characterized members in DUF1887 family

(**A**) Plasmid challenge assay of *E. coli* carrying plasmids encoding Csm, Cdn1, and corresponding mutants described in methods. (**B**) Structural alignment of the active states of TsCard1 and Cdn1. (**C**) Structure-based sequence alignment of DUF1887 family proteins, categorized into Cdn1-like, TsCard1-like, and StCan2-like subgroups. Different clades and corresponding species are annotated on the left. (**D**) Sequence logos illustrating highly conserved motifs unique to the Cdn1-like clade.

Positions are numbered according to the reference protein. (**E**) Structural superimposition of the cA_4 binding pockets in Cdn1, TsCard1, and StCan2, shown in cartoon representation. (**F**) Surface representation of the cA_4 binding pocket in Cdn1, TsCard1, and StCan2, viewed from the bottom of CARF domain.

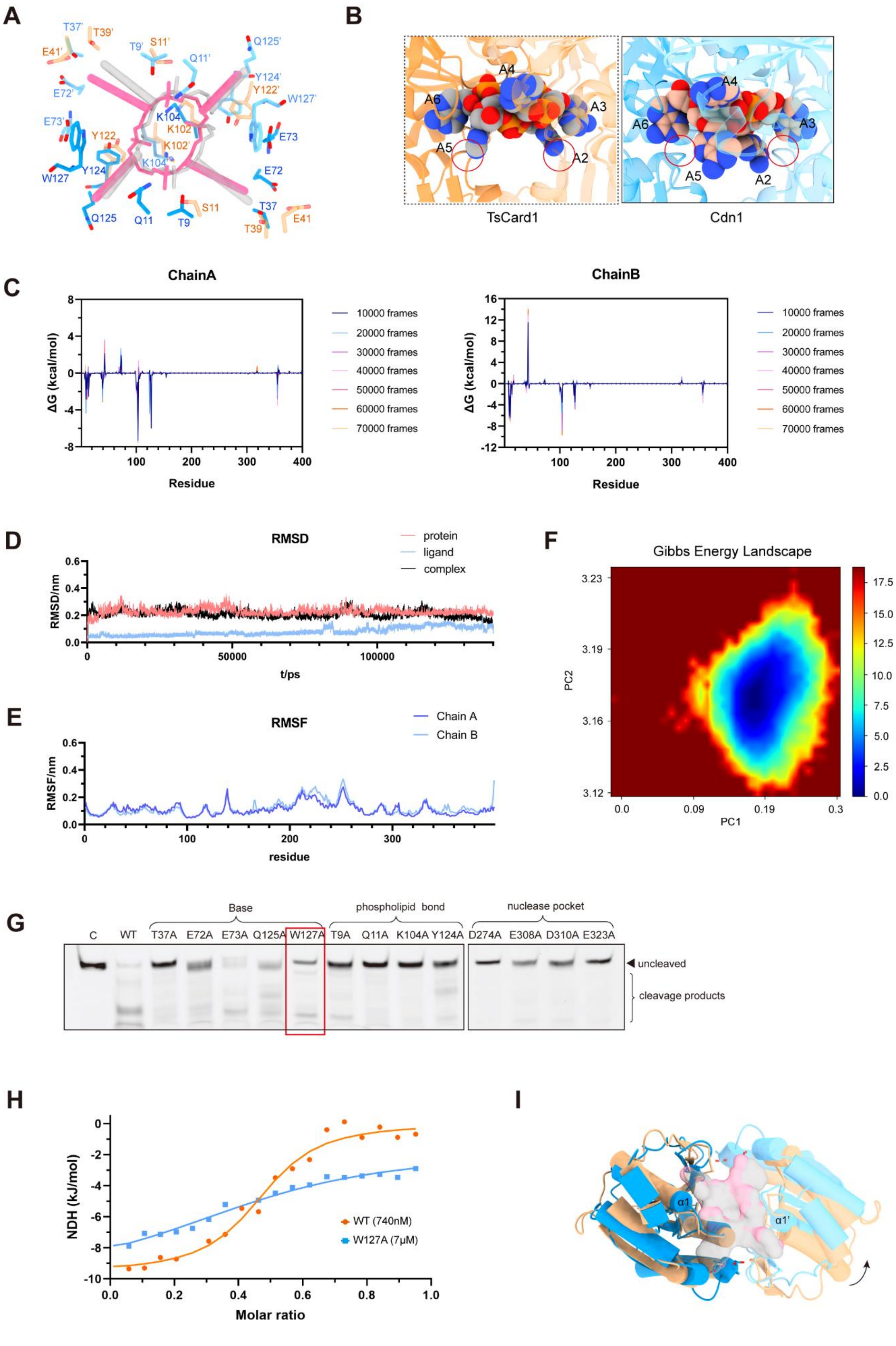
A
T37'
T39'
T9'
S11'
Q125'
E41'
Q11'
Y124'
E72'
Y122'
W127'
E73'
K104
K102
Y122
E73
K102'
K104'
E72
W127
Y124
T37
Q125
Q11
T9
S11
T39
E41
B
A4
A6
A3
A5
A2
TsCard1
Cdn1
C
ChainA
ChainB
ΔG (kcal/mol)
Residue
10000 frames
20000 frames
30000 frames
40000 frames
50000 frames
60000 frames
70000 frames
D
RMSD
protein
ligand
complex
RMSD/nm
t/ps
E
RMSF
Chain A
Chain B
RMSF/nm
residue
F
Gibbs Energy Landscape
PC1
PC2
G
Base
phospholipid bond
nuclease pocket
C
WT
T37A
E72A
E73A
Q125A
W127A
T9A
Q11A
K104A
Y124A
D274A
E308A
D310A
E323A
uncleaved
cleavage products
H
NDH (kJ/mol)
Molar ratio
WT (740nM)
W127A (7μM)
I
α1
α1'

Supplementary Figure.7 MD simulation results and structural comparison between modeled cA_6-Cdn1 and cA_6-TsCard1

(**A**) Structural comparison of the cA_4-binding pocket between cA_4-Cdn1 and cA_4-TsCard1. In the cA_4-TsCard1 structure, cA_4 and the protein are colored in gray and orange, respectively, while in the cA_4-Cdn1 structure, cA_4 and the protein are colored in pink and blue, respectively. (**B**) Comparison of steric clashes and the absence of clashes between cA_6 and loop residues in TsCard1 or Cdn1, shown in space-filling representation. (**C**) Estimation of the binding free energy of the complex cA_6-Cdn1 based on the GBSA method, with sampling intervals of 10000 to 70000 frames. (**D**) RMSD of of cA_4-Cdn1 complex model for 140ns MD simulation. (**E**) RMSF of both protein chains and ligand chain in the MD simulation. (**F**) Gibbs energy landscape generated using Dulvty for cA_4-Cdn1. (**G**) Effects of key residues on cA_6-induced activation. The W127A mutant is highlighted with a red rectangle. (**H**) ITC results demonstrating the importance of W127A for the binding between cA_6 and Cdn1. (**I**) Superimposition of cA_4-Cdn1 and cA_4-TsCard1, aligned through one monomer (blue and salmon). The α1 helix of both Cdn1 chains are labeled, with the rotation direction indicated by an arrow. Key residues E72 and E73 of Cdn1 are shown as sticks representations, colored by heteroatom.

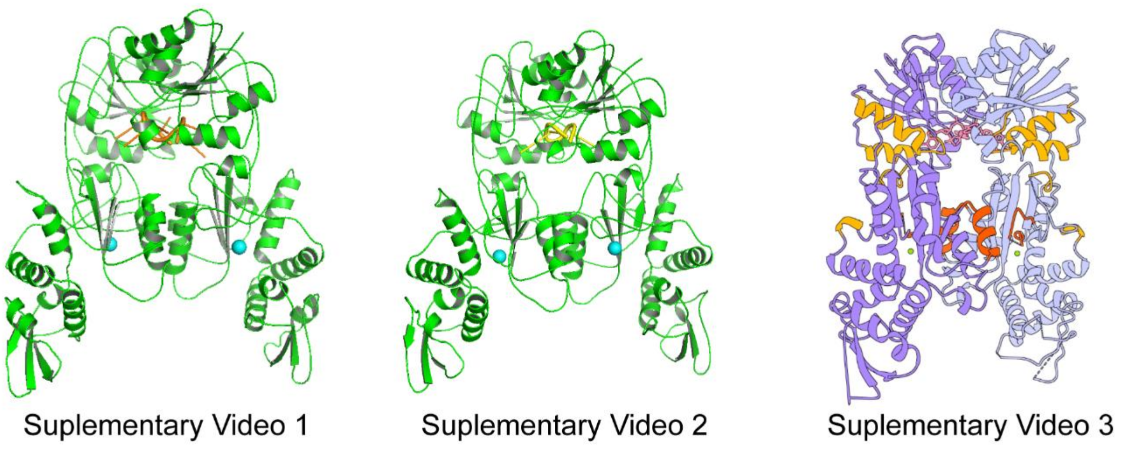

Supplementary Video 1. The trajectory representation of cA_6-Cdn1 complex during 150ns MD simulation

Cdn1 is colored in green, cA_6 is colored in orange, and Mg^{2+} are colored in cyan.

Supplementary Video 2. The trajectory representation of cA_4-Cdn1 complex during 150ns MD simulation

Cdn1 is colored in green, cA_4 is colored in yellow, and Mg^{2+} are colored in cyan.

Supplementary Video 3. Conformational changes between inactive and active states

The transition between the inactive and active states is illustrated. cA_4 is colored pink and is initially displayed slowly to represent its binding process. Key residues involved in conformational changes (residues 7–25, 151–158, and 169–171) are highlighted in orange. In the active state, helix 369–379 and loop 300–308 at the dimeric nuclease interface are shown in orange-red, along with the appearance of green magnesium ions at the end of the video.
